# N-myristoyltransferase-1 is necessary for lysosomal degradation and mTORC1 activation in cancer cells

**DOI:** 10.1038/s41598-020-68615-w

**Published:** 2020-07-20

**Authors:** Yu-Chuan Chen, Marian S. Navarrete, Ying Wang, Natalie C. McClintock, Reiko Sakurai, Feng Wang, Kathryn T. Chen, Tsui-Fen Chou, Virender K. Rehan, Delphine J. Lee, Begoña Diaz

**Affiliations:** 1 Division of Medical Hematology Oncology, Department of Medicine, The Lundquist Institute for Biomedical Innovation at Harbor-UCLA Medical Center, Torrance, CA 90502 USA; 2 Division of Dermatology, Department of Medicine, The Lundquist Institute for Biomedical Innovation at Harbor-UCLA Medical Center, Torrance, CA 90502 USA; 3 Division of Neonatology, Department of Pediatrics, The Lundquist Institute for Biomedical Innovation at Harbor-UCLA Medical Center, Torrance, CA 90502 USA; 4 Division of Surgical Oncology, Department of Surgery, Harbor-UCLA Medical Center, Torrance, CA 90502 USA; 5 Division of Medical Genetics, Department of Pediatrics, The Lundquist Institute for Biomedical Innovation at Harbor-UCLA Medical Center, Torrance, CA 90502 USA; 6 David Geffen School of Medicine at UCLA, Los Angeles, CA USA; 7 Jonsson Comprehensive Cancer Center, UCLA, Los Angeles, CA USA; 8 Present Address: Biology and Biological Engineering, California Institute of Technology, Pasadena, CA USA

**Keywords:** Cancer, Cell biology

## Abstract

N-myristoyltransferase-1 (NMT1) catalyzes protein myristoylation, a lipid modification that is elevated in cancer cells. NMT1 sustains proliferation and/or survival of cancer cells through mechanisms that are not completely understood. We used genetic and pharmacological inhibition of NMT1 to further dissect the role of this enzyme in cancer, and found an unexpected essential role for NMT1 at promoting lysosomal metabolic functions. Lysosomes mediate enzymatic degradation of vesicle cargo, and also serve as functional platforms for mTORC1 activation. We show that NMT1 is required for both lysosomal functions in cancer cells. Inhibition of NMT1 impaired lysosomal degradation leading to autophagy flux blockade, and simultaneously caused the dissociation of mTOR from the surface of lysosomes leading to decreased mTORC1 activation. The regulation of lysosomal metabolic functions by NMT1 was largely mediated through the lysosomal adaptor LAMTOR1. Accordingly, genetic targeting of LAMTOR1 recapitulated most of the lysosomal defects of targeting NMT1, including defective lysosomal degradation. Pharmacological inhibition of NMT1 reduced tumor growth, and tumors from treated animals had increased apoptosis and displayed markers of lysosomal dysfunction. Our findings suggest that compounds targeting NMT1 may have therapeutic benefit in cancer by preventing mTORC1 activation and simultaneously blocking lysosomal degradation, leading to cancer cell death.

## Introduction

Myristoylation consists of the transfer of the fatty acid myristic acid to proteins^1^, and is catalyzed in vertebrates by two isoenzymes called N-myristoyltransferases (NMTs): NMT1^2^ and NMT2^3^. Myristoylation increases the affinity of proteins for plasma and internal cell membranes and modulates protein activity by a number of mechanisms^1,4^. NMT1 is essential for early embryonic development^5^, and deletion of both isoenzymes impairs T cell development and activation in mice^6^. NMT1 expression is increased in cancer^7^, associates with poor patient outcome^8^, and has been considered as a target for therapeutic intervention^9^. Accordingly, NMT1 activity promotes proliferation and survival of ovarian and colon cancer cells^10^, facilitates cancer cells survival through AMPKβ-dependent regulation of mitophagy^8^, promotes Src-dependent cell cycle progression in prostate cancer^11^, and regulates proteostasis^12,13^.

Lysosomes are complex multifunctional organelles with an essential role in cellular metabolism^14^. Lysosomal hydrolases convert biological macromolecules into monomeric forms that can be catabolized to generate energy^15^. Extracellular cargo is internalized through endocytosis^16,17^, whereas intracellular cargo derives from macroautophagy (autophagy hereinafter), in which cellular macromolecules or organelles are routed to digestion^18,19^. Lysosomes have also a central role in nutrient sensing and anabolism^14,15^. Amino acid sensing and full activation of mTORC1 by amino acids and growth factors occurs at the lysosomal surface, where mTORC1 is tethered by a multiprotein complex named RAGULATOR^20–25^. The binding of RAGULATOR to the lysosome is mediated by lipidation (myristoylation and palmitoylation) of its member LAMTOR1 (late endosomal/lysosomal adaptor, MAPK and mTOR activator 1)^21,26^. mTORC1 facilitates protein, lipid, and nucleic acid synthesis, promoting cell growth and supporting cell proliferation^27–32^.

Because the coordination of catabolic and anabolic lysosomal functions is critical to maintain metabolic homeostasis, a lysosomal autoregulatory mechanism is in place to increase lysosomal abundance in response to starvation through the activity of transcription factors TFEB and TFE3. In the presence of nutrients, TFEB and TFE3 are localized on the lysosomal surface through direct mTORC1 phosphorylation. Nutrient depletion and mTORC1 inactivation leads to de-phosphorylation, nuclear translocation, and transcriptional activation of a network of genes involved in lysosomal biogenesis and autophagy^33–36^.

Oncogenic transformation is associated with elevated lysosomal abundance and activity, essential to maintain the high metabolic demands and increased proliferation of cancer cells^37–39^. Lysosomal-associated transcriptional activity of TFEB and TFE3 is aberrantly activated in some cancers through constitutive nuclear localization, which overrides the brake imposed by nutrient availability on lysosomal functions^40,41^. Due to their dependency of lysosomal activity, cancer cells are particularly sensitive to perturbations in lysosomes. Because blocking a single lysosomal metabolic function (for instance by treatment with chloroquine—which inhibits degradation—or with mTOR inhibitors) often result in cellular adaptation, compounds that simultaneously block lysosomal catabolic and anabolic functions are regarded as promising anti-cancer strategies^42^.

Here, we report an unexpected essential role for NMT1 in the regulation of lysosomal degradation and mTORC1 activation in cancer cells. Inhibition of NMT using a small compound decreased cancer cell viability in vitro and in vivo through inhibition of mTORC1 and simultaneous blockade of lysosomal degradation, mostly through inactivation of the lysosomal adaptor LAMTOR1. Our findings uncover an additional function for NMT1 in cellular homeostasis, and a novel druggable lysosomal metabolic vulnerability of cancer cells.

## Results

### Targeting NMT1 slowed the autophagy flux in cancer cells

**Figure 1:**
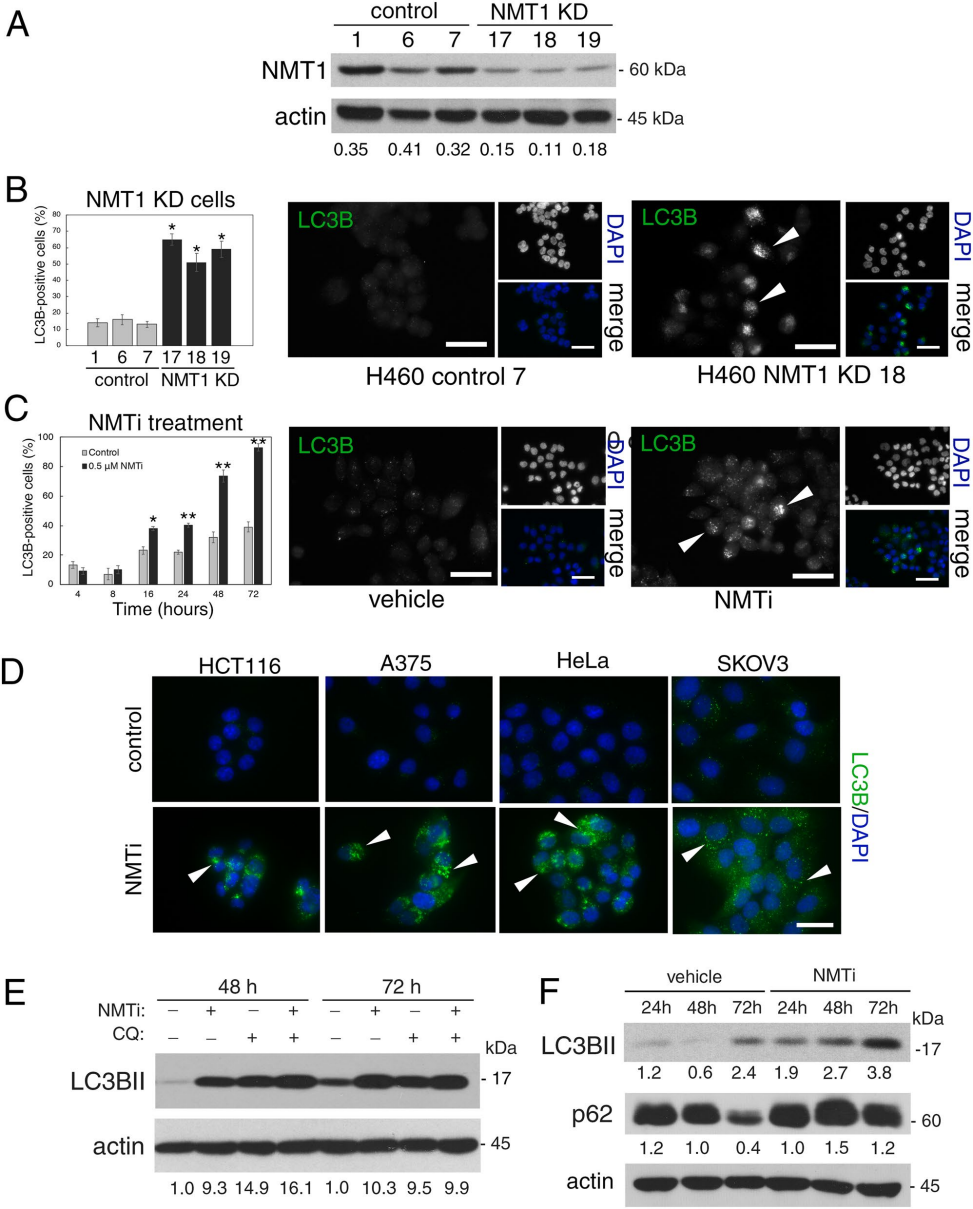
NMT1 activity is necessary to maintain the autophagic flux in cancer cells. (**A**) NMT1 expression in control and NMT1 KD H460 clones. Numbers under each lane, densitometry values (arbitrary units) for NMT1 signal normalized to actin in one representative experiment from two independent experiments. (**B**) LC3B IF in control and NMT1 KD H460 lines. DAPI, nuclei. Graph: average percent of LC3B-positive cells from two independent experiments combined. At least 100 cells per condition and experiment were analyzed. Error bars, SEM. *p < 0.005 (Student’s *t* test) with respect to controls. Right, representative LC3B staining in H460 control and NMT1 KD lines. DAPI, nuclei. (**C**) LC3B IF in H460 cells treated with DMSO (vehicle) or 0.5 μM NMTi for the indicated times. DAPI, nuclei. Graph: average percent of LC3B-positive cells from three independent experiments combined. At least 80 cells per condition and experiment were analyzed. Error bars, SEM. *p < 0.0005, **p < 0.0001 (Student’s *t* test). Right, representative pictures of LC3B and DAPI staining of H460 cells treated with DMSO or NMTi for 48 h. (**D**) LC3B IF in the indicated cancer cell lines treated with DMSO (vehicle) or NMTi for 72 h. DAPI, nuclei. The experiment was repeated twice with similar results. (**E**) H460 cells treated with 1 μM NMTi for the indicated times in culture and/or with 30 μM chloroquine (CQ) for 3 h prior to processing for WB using an LC3B antibody. Actin was used as loading control. Numbers under each lane are densitometry values (arbitrary units) for LC3BII signal normalized to actin and relative to the corresponding 24 h time point. One representative experiment from thee independent experiments is shown. (**F**) H460 cells treated with 0.5 μM NMTi for the indicated times in culture. WB results for LC3BII, p62^SQSTM^ and actin (loading control) are shown for one representative experiment from two independent experiments with similar results. Numbers under each blot are densitometry values (arbitrary units) for LC3BII and p62^SQSTM^ signal normalized to actin. Arrowheads, autophagic vesicles. Bar, 4 μm in (**B**), (**C**) and 3 μm in (**D**).

NMT1 facilitates mitophagy in cancer cells^8^, but its role in general autophagy is not known. We generated H460 (non-small cell lung carcinoma) cells with reduced levels of NMT1 using lentiviral transduction of an NMT1-specific shRNA targeting sequence, followed by clonal selection in the presence of puromycin. Control and knock-down (KD) clones generated in parallel were selected for further analysis. Western blotting (WB) revealed that NMT1 protein expression was reduced between 50 and 60% (Fig. 1A), and no compensatory induction of NMT2 expression was observed in KD clones (Supplementary Fig. S1). Immunofluorescence (IF) for LC3B, a component of autophagosome membranes, revealed a 2–3 fold increase in the percent of cells containing LC3B-positive puncta in all NMT1 KD clones when compared with control clones (Fig. 1B). We verified these results using different targeting sequences by transiently transfecting an NMT1 siRNA pool in H460 cells (Supplementary Fig. S1).

To achieve pharmacological inhibition of NMT1, we used the *Trypanosoma brucei* NMT inhibitor DDD85646^43^, which also inhibits human NMT1 and 2 with high potency^44^, and has been validated as a highly specific NMT inhibitor^45^ . Previous studies in HeLa cells found decreased myristoylation after treatment with 0.5–1 μM DDD85646^44^ (referred to as NMTi hereinafter). We confirmed that 1 μM NMTi effectively decreased global myristoylation in H460 cells (Supplementary Fig. S1), and used NMTi at a concentration between 0.5 and 1 μM for subsequent experiments. Time-course experiments using NMTi treatment in H460 and H1792 cells (lung adenocarcinoma) revealed a time-dependent increase in the fraction of cells containing LC3B-positive puncta (Fig. 1C and Supplementary Fig. S1). Accumulation of LC3B-positive puncta was also observed in colon (HCT116), melanoma (A375), cervical (HeLa) and ovarian (SKOV3) cancer cells treated with NMTi (Fig. 1D).

Elevated autophagosome content may be the result of increased autophagy or decreased autophagic flux. To differentiate between the two, we combined NMTi treatment with chloroquine (CQ), an inhibitor of lysosomal degradation that efficiently blocks the autophagic flux. Whereas treatment of H460 cells with CQ or NMTi alone led to comparable levels of LC3BII accumulation, the combination treatment did not show an additive effect (Fig. 1E). This indicated that the accumulation of LC3BII after NMT inhibition is mostly due to impairment of the autophagy flux.

Consistent with nutrient depletion over time in culture, untreated cells had a time-dependent increase in LC3BII-positive puncta by IF (Fig. 1C), and an increase in LC3BII abundance by WB (Fig. 1E). Accordingly, total levels of the autophagosome adaptor p62^SQSTM^, which is degraded in the autolysosome during normal autophagy^46^, decreased as LC3BII levels increased in H460 cells (Fig. 1F) and H1792 cells (Supplementary Fig. S1). This was in contrast with cells treated with NMTi, in which the abundance of p62^SQSTM^ remained elevated despite increased accumulation of LC3BII (Fig. 1F and Supplementary Fig. S1), supporting the idea that NMTi treatment impairs the autophagy flux in cancer cells. IF of H1792 cells with a p62^SQSTM^ antibody confirmed these results by revealing increased abundance of p62-positive puncta in cells treated with NMTi for 72 h (5.6 ± 1.3 per cell in control vs. 37.7 ± 4.1 in NMTi treated cells (Supplementary Fig. S1).

### Inhibition of NMT1 decreased lysosomal degradation and caused accumulation of late endosome/lysosomes

**Figure 2:**
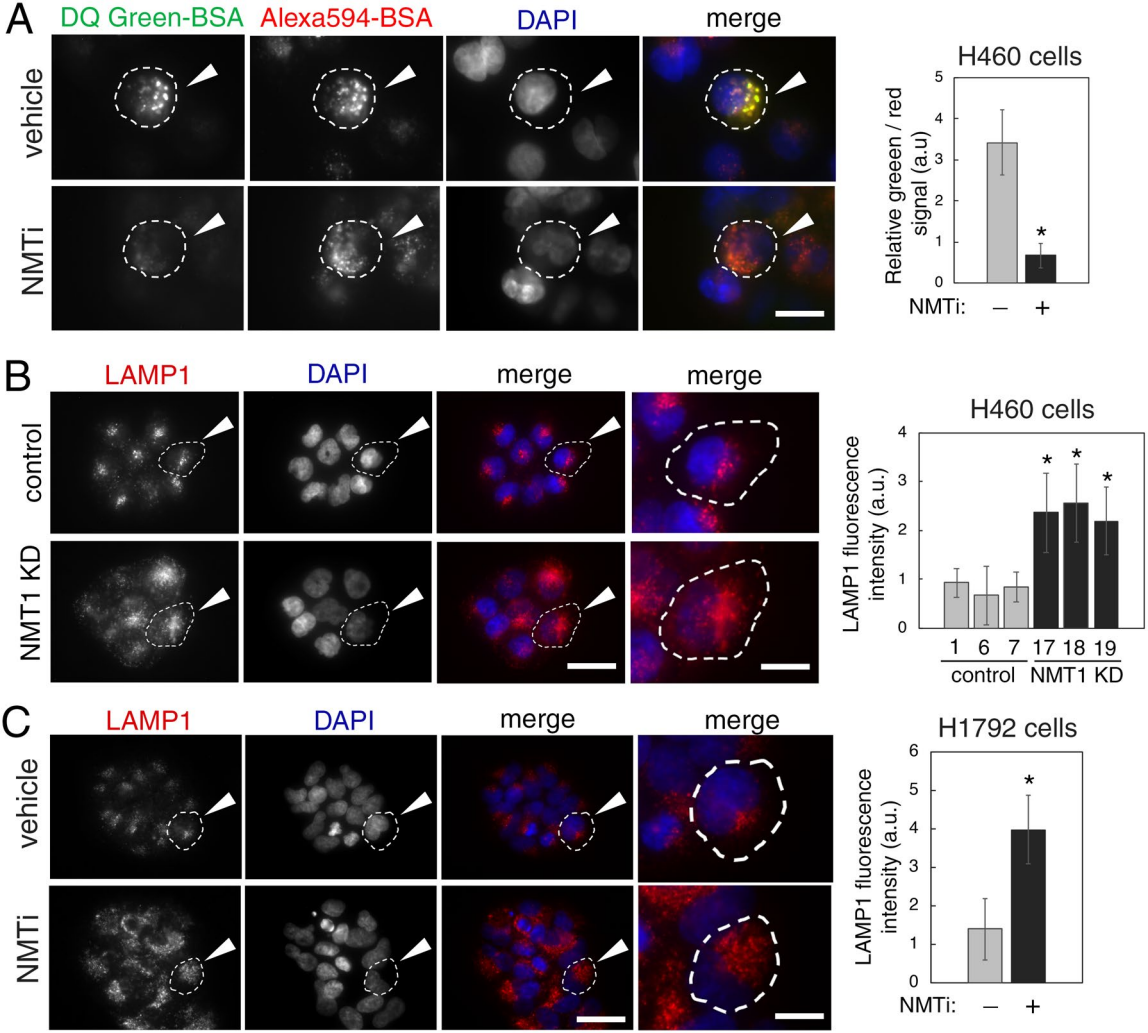
NMT1 targeting decreased lysosomal degradation and increased lysosomal abundance in cancer cells. (**A**) H460 cells were incubated with AlexaFluor594-BSA and DQ-Green-BSA for 18 h and then treated with 1 μM NMTi or DMSO (vehicle) for 8 h. After fixation and mounting, cells were imaged to detect BSA uptake (red fluorescence from Alexa 594-BSA) and BSA degradation (green fluorescence from cleaved DQ-Green BSA). DAPI was used to label nuclei. Images show representative results of vehicle and NMTi treated cells. Arrowheads (top), cell containing green fluorescence, indicative of intact lysosomal degradative activity. Arrowheads (bottom), cell containing only red fluorescence, indicative of impaired lysosomal degradation despite efficient uptake. Right, lysosomal degradation was quantified in three independent experiments (at least 50 cells per condition and experiment), and represented as average and SEM of relative green vs. red fluorescence. *p < 0.01 (Student’s *t* test). (**B**) Representative images of lysosome/late endosome detection by LAMP1 IF in H460 control and NMT1 KD cell lines. DAPI was used to stain nuclei. Arrowheads, representative cell outlined and magnified in the last column. Right, quantification of LAMP1-positive signal per cell from two independent experiments combined. At least 25 cells were analyzed per condition and experiment. Graph shows average ± SEM. *p < 0.01 (Student’s *t* test) for all KD clones with respect to each of the control clones. (**C**) H1792 cells were treated with 0.5 μM NMTi or DMSO control for 72 h and processed for LAMP1 IF. DAPI was used to label nuclei. Images show representative results. Arrowheads, representative outlined cell magnified in the last column. Right, quantification of LAMP1-positive signal per cell from two independent experiments combined. At least 20 cells were analyzed per condition and experiment. Graph, average ± SEM. *p < 0.01 (Student’s *t* test). Bar, 1 μm in (**A**); 4 μm and 1 μm in (**B**) and (**C**) (first 3 and last column respectively).

Lysosomes are the final destination of autophagosome cargo, where intracellular biological macromolecules and organelles are enzymatically degraded. Because defective lysosomal degradation blocks the autophagy flux, we evaluated the ability of cancer cells treated with NMTi to degrade internalized cargo in lysosomes. We labeled cells with two bovine serum albumin (BSA) probes to monitor lysosomal degradation: DQ-green BSA, which emits fluorescence only after proteolytic degradation at lysosomes, and AlexaFluor 549 BSA, which is constitutively fluorescent and served as a control for probe internalization. Incubation with both BSA probes in the presence of NMTi treatment revealed decreased lysosomal degradation of DQ-green BSA despite effective internalization of AlexaFluor 549 BSA (Fig. 2A). This indicated that NMT1 is necessary for effective lysosomal degradation in cancer cells, in line with the observed delay in the autophagy flux, and the accumulation of p62^SQSTM^ in cells treated with NMTi.

We used IF with an antibody against the lysosome-associated membrane protein 1 (LAMP1), a marker of late endosomes and lysosomes, to analyze the lysosomal compartment in cells with reduced NMT1. When compared with control lines, NMT1 KD lines had a 40–50% increase in LAMP1-fluorescence intensity (Fig. 2B), which was also detected in H1792 cells transfected with an NMT1 siRNA pool (Supplementary Fig. S2). Treatment with NMTi also led to the accumulation of LAMP1-positive vesicles in H1792 (Fig. 2C), as well as in HCT116, A375, HeLa and SKOV3 cancer cells (Supplementary Fig. S2). These results suggest increased lysosomal biogenesis in cells lacking NMT1 activity.

### Increased apoptosis and nuclear accumulation of TFE3 in cancer cells with decreased NMT1 activity

**Figure 3:**
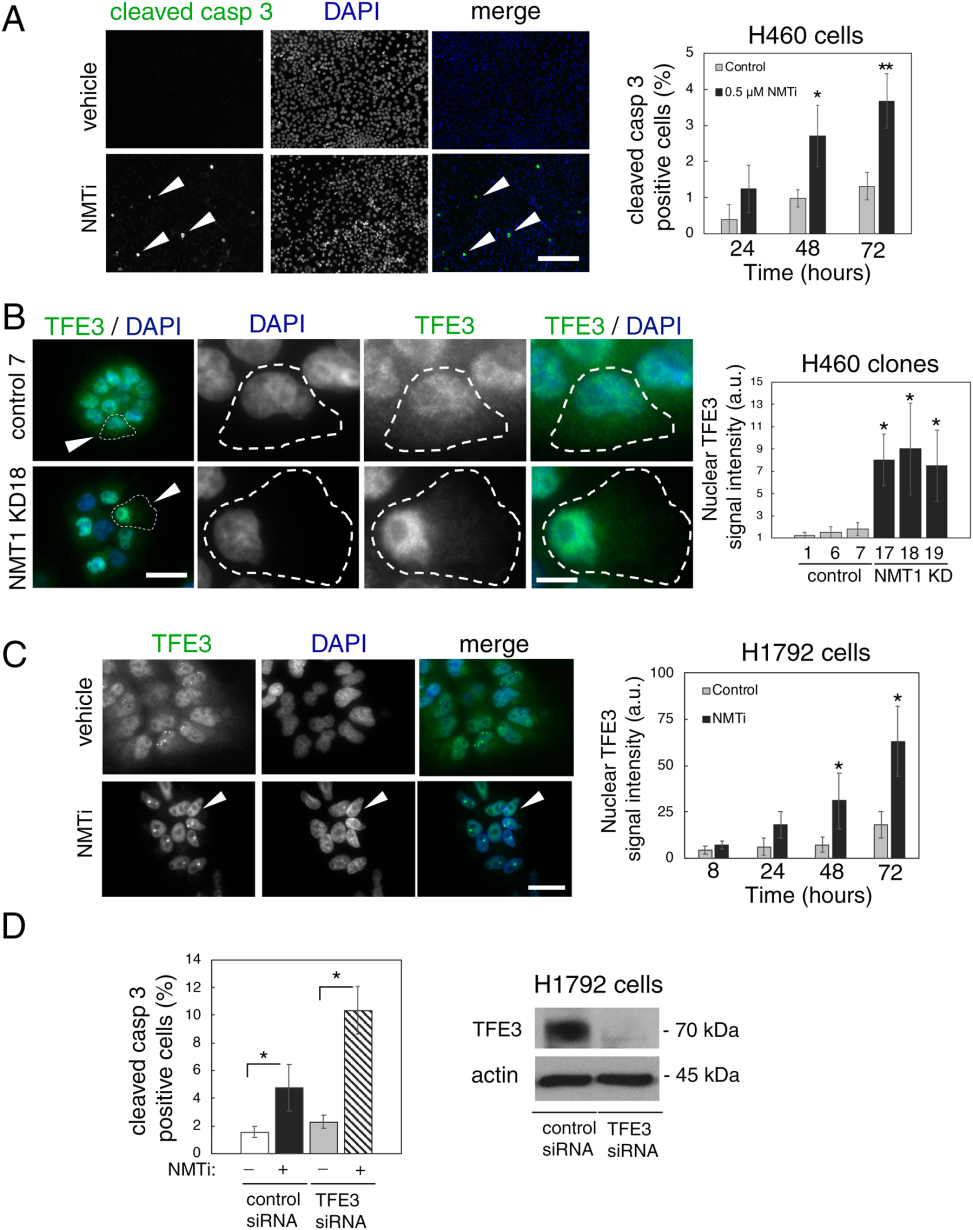
NMT1 targeting induced apoptosis and nuclear accumulation of TFE3 in cancer cells. (**A**) H460 cells treated with 0.5 μM NMTi or DMSO (vehicle) for the indicated periods of time stained with cleaved caspase 3 antibody to detect apoptotic cells. DAPI was used to label nuclei. Left, representative images from cells treated for 72 h. Arrowheads, cleaved caspase 3-positive cells. Percent of positive cells was quantified from three independent experiments. At least 60 cells were analyzed per condition and experiment. Graph shows results from combined experiments as average ± SEM. *p < 0.05, **p < 0.001 (Student’s *t* test). (**B**) H460 control and NMT1 KD lines stained with TFE3 antibody and DAPI to detect nuclei. Left, representative images. Arrowheads in column 1 indicate cells magnified in pictures from column 2 to 4. Intensity of nuclear TFE3 signal was quantified in two independent experiments, and at least 30 cells were analyzed per condition and experiment. Graph shows data from combined experiments as average ± SEM. *p < 0.01 (Student’s *t* test). (**C**) H1792 cells treated with 0.5 μM NMTi or DMSO (control) for the indicated times, stained with a TFE3 antibody and DAPI. Left, representative images from H1792 cells treated for 72 h. Arrowheads, cell with strong nuclear TFE3 signal. Right, quantification of nuclear TFE3 signal intensity at each time point represented as average ± SEM. *p < 0.001, **p < 0.0001 (Student’s *t* test) with respect to their relative DMSO control for one representative experiment out of two independent experiments with similar results. (**D**) H1792 cells transfected with non-targeting control or TFE3 siRNA for 72 h and treated with 0.5 μM NMTi or DMSO control for 48 h. Cells were stained with cleaved caspase 3 antibody and DAPI. Quantification of apoptotic cells (at least 50 cells per condition and experiment) from two independent experiments combined is represented as average ± SEM. *p < 0.01 (Student’s *t* test). WB for with TFE3 in H1792 cell at 72 h after transfection is shown. Actin was used as a loading control. Bar, 100 μm in (**A**), 5 μm in (**B**) and (**C**).

Treatment of H460 cells with NMTi for 48 h or longer induced apoptosis as detected by cleaved caspase 3 staining (Fig. 3A), a finding in agreement with previous reports in other cancer lines^12^. The transcription factor TFE3 responds to lysosomal dysfunction by nuclear translocation and transcriptional activation of genes involved in lysosomal biogenesis as a pro-homeostatic and pro-survival mechanism^34,40^. To analyze whether targeting NMT1 affects TFE3 subcellular distribution, we stained H460 NMT1 KD clones with a TFE3 specific antibody. Consistent with previous reports in pancreatic cancer^41^, we detected both cytoplasmic and nuclear TFE3 localization in H460, H1792 and HeLa cells (Fig. 3B,C and Supplementary Fig. S3). Decreased NMT1 levels further increased the nuclear localization of endogenous TFE3 in all H460 KD clones (Fig. 3B). Similarly, treatment with NMTi caused a time-dependent increase in the nuclear accumulation of endogenous TFE3 in H1792 (Fig. 3C), and in HeLa cells (Supplementary Fig. S3). Next, we generated pools of H1792 and H460 stably expressing TFE3-GFP, and subjected them to NMTi treatment. Using these overexpression assay, we confirmed that TFE3-GFP was both cytoplasmic and nuclear in most cells, and that TFE3-GFP further accumulated in the nucleus after 72 h of NMTi treatment (86.5 ± 4.3% treated vs. 15.4 ± 2.6% control for H1792 TFE3-GFP cells and 92.5 ± 4.4% treated vs. 14.7 ± 2.6% control for HeLa TFE3-GFP cells in one representative experiment, see Supplementary Fig. S3).

To investigate whether TFE3 plays a functional role in the pro-apoptotic effects of NMTi treatment, we used a TFE3 specific siRNA pool to decrease endogenous TFE3 expression, and treated cells 24 h after transfection with NMTi for 48 h. Staining with cleaved caspase 3 revealed that H1792 cells with reduced levels of TFE3 were about 2-fold more sensitive to NMTi-induced apoptosis than control cells, but did not show increased apoptosis in vehicle-treated cells (Fig. 3D and Supplementary Fig. S3). This finding suggests that TFE3 nuclear accumulation is part of a pro-survival response to lysosomal dysfunction in cancer cells treated with NMTi.

### Inhibition of NMT1 decreased mTORC1 activation and proliferation of cancer cells

**Figure 4:**
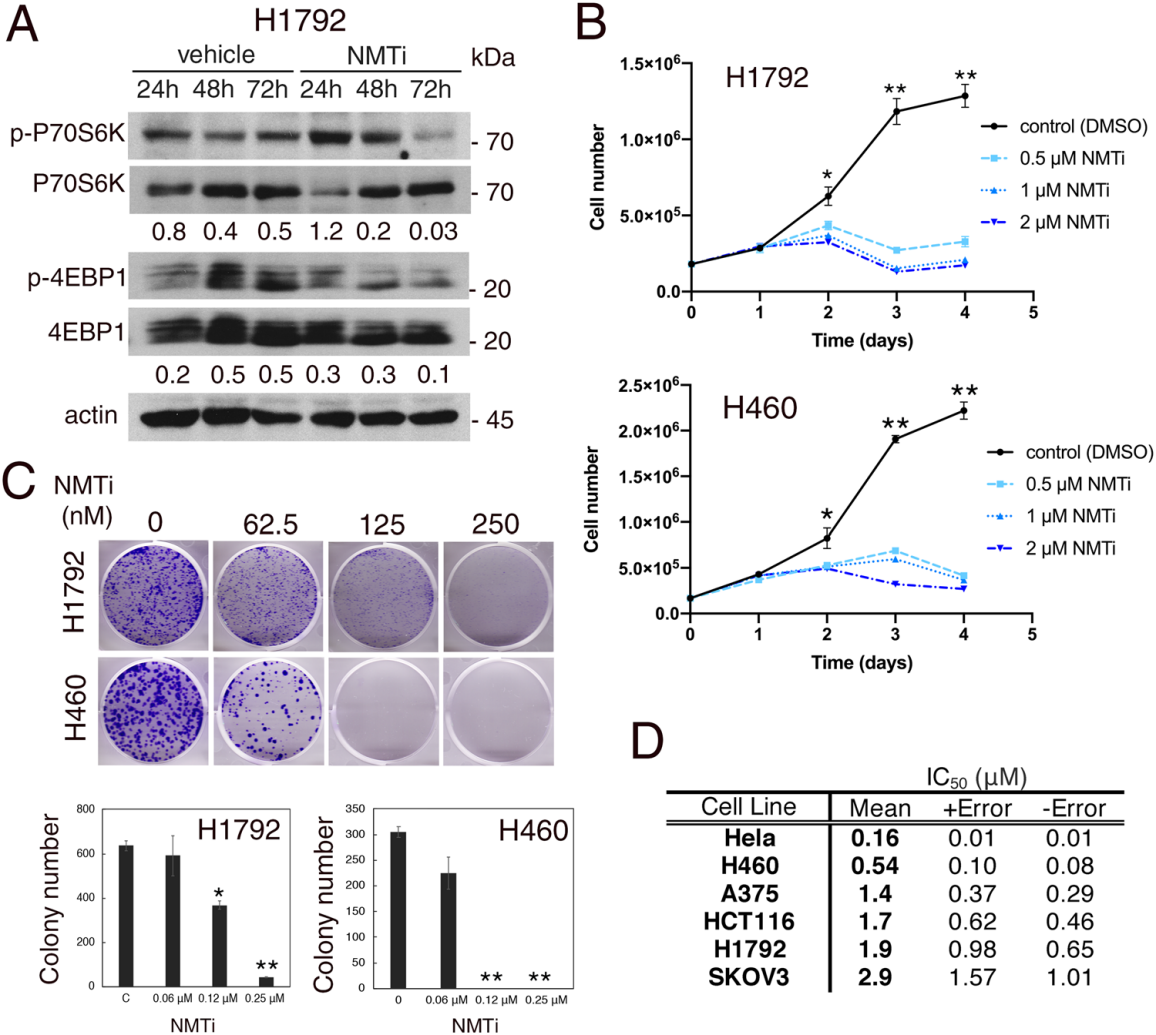
Inhibition of NMT decreased mTORC1 activation, proliferation and colony forming ability of cancer cells. (**A**) WB analysis of H1792 cells treated with 0.5 μM NMTi for the indicated times using antibodies against p(T389)p70S6K and p(T37/43)4E-BP1. Antibodies against total p70S6K and 4EB-P1 were used for normalization, and actin was used as loading control. Numbers under each lane are densitometry values (arbitrary units) for phospho- vs. total signal for each protein normalized to actin, in one representative experiment (from two independent experiments with similar results). Uncropped membrane scans are shown in Supplementary Figure S8. (**B**) Proliferation curves for H1792 and H460 cells treated with the indicated concentrations of NMTi for the indicated periods of time (one representative experiment of three independent experiments per cell line is shown). Total number of viable cells per plate for each time point and condition are represented as average ± SEM. *p < 0.01 for 0.5 μM vs. control, and p < 0.005 for 1 and 2 μM vs. control. **p < 0.000001 (Student’s *t* test) for treated (all dosages) vs. control. (**C**) H1792 and H460 cells were treated with the indicated concentrations of NMTi for 7–10 days and colonies stained with crystal violet. Pictures, scans of representative wells. Three wells per condition were quantified in three independent experiments per cell line with similar results. Bottom, quantification of colony number per well in one representative experiment, represented as average ± SEM. *p < 0.05, **p < 0.0001 (Student’s *t* test) for treated vs. control. (**D**) NMTi IC_50_ values were calculated at 72 h of treatment in the indicated cell lines using Cell Titer Glo assay.

TFE3 is retained on the lysosomal surface through direct phosphorylation by the mTORC1 complex, and translocates to the nucleus upon mTORC1 inhibition. Accumulation of nuclear TFE3 after NMTi treatment suggests that NMT1 inhibition causes mTORC1 inactivation. To test this hypothesis, we analyzed the phosphorylation status of the mTORC1 targets P70S6K and 4EB-P1 using p(T389)P70S6K and p(T37/43)4EB-P1 specific antibodies. We treated H460 and H1792 cells with NMTi for 24–72 h and analyzed cell lysates using WB. Phosphorylation of 4EB-P1 decreased after 48 h of treatment, and phosphorylation of P70S6K decreased after 72 h in both lines when compared with control lysates (Fig. 4A and Supplementary Fig. S4). This indicated that NMT1 activity is necessary to maintain basal levels of mTORC1 activation in cancer cells. Consistent with the role of mTORC1 at sustaining proliferation, NMTi treatment decreased H1792 and H460 cell proliferation in a dose and time-dependent manner (Fig. 4B), and H460 NMT1KD clones (with about 50% reduction in NMT1 expression, Fig. 1A), proliferated more slowly than their corresponding control lines (Supplementary Fig. S4). Furthermore, NMTi treatment decreased the colony-forming ability of H460 and H1792 cells in a dose-dependent manner (Fig. 4C), and had a similar effect in HCT116, A375, HeLa and SKOV3 cells (Supplementary Fig. S4). The NMTi IC_50_ values for these cells, analyzed using a viability test assay, were between 0.16 and 2.9 μM (Fig. 4D).

### NMT1 targeting prevented lysosomal localization of LAMTOR1 and mTOR

**Figure 5:**
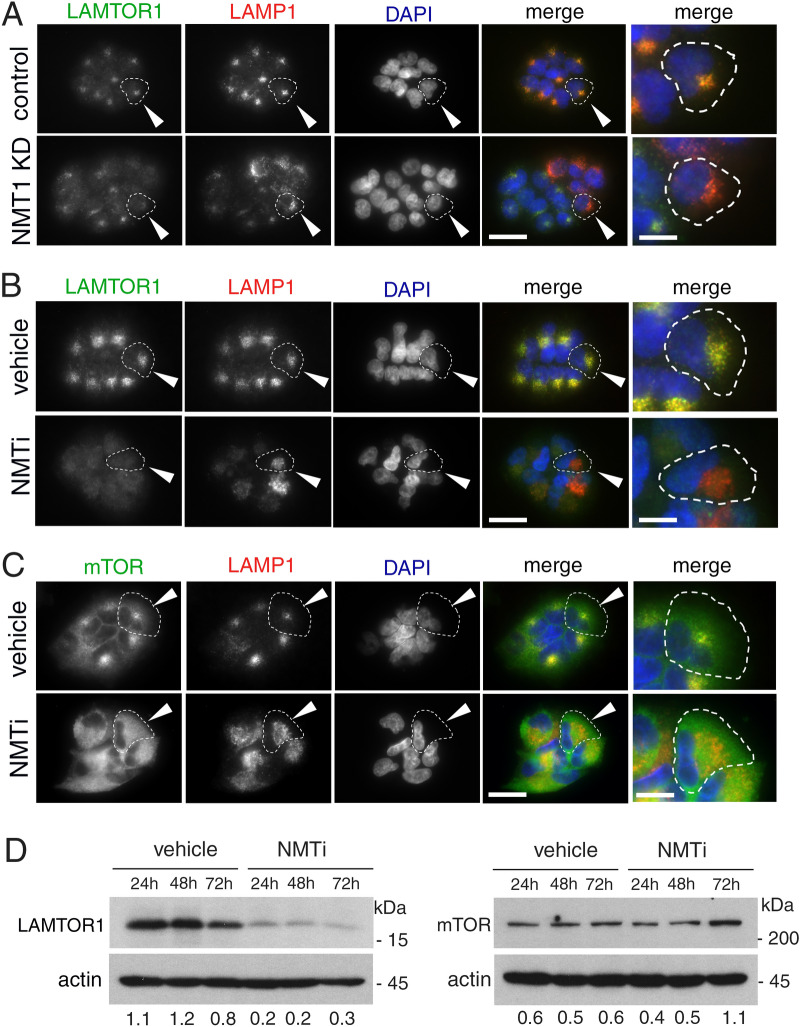
Targeting NMT1 impaired lysosomal localization of LAMTOR1 and mTOR and decreased LAMTOR1 protein levels. (**A**) H460 control and NMT1 KD lines were stained with LAMTOR1 and LAMP1 antibodies along with DAPI to detect nuclei. Panel shows representative fluorescence microscopy images of the indicated control and NMT1 KD clones. Three independent experiments were performed with similar results. Arrowhead, outlined representative cell magnified in the last column. (**B**) H1792 cells were treated with 0.5 μM NMTi or DMSO control (vehicle) for 48 h, and stained with LAMTOR1 and LAMP1 antibodies. DAPI was used to stain nuclei. Panel shows representative fluorescence microscopy images from two independent experiments with similar results. Arrowhead, outlined representative cell magnified in the last column. (**C**) H1792 cells were treated with 0.5 μM NMTi or DMSO control (vehicle) for 48 h, and stained with mTOR and LAMP1 antibodies. DAPI was used to stain nuclei. Panel shows representative fluorescence microscopy images from two independent experiments with similar results. Arrowhead, outlined representative cell magnified in the last column. (**D**) Protein lysates from H1792 cells treated with 0.5 μM NMTi or DMSO (control) for the indicated times were immunoblotted for LAMTOR1 and mTOR. Actin was used as loading control. Numbers under each lane are densitometry values (arbitrary units) for LAMTOR1 or mTOR signal normalized to actin in one representative experiment from two independent experiments with the same result. Uncropped scans are shown in Supplementary Figure S8. Bars, 5 μm in (**A**)–(**C**) columns 1–4, and 1 μm in last column.

Because myristoylation of the RAGULATOR complex member LAMTOR1 is necessary for lysosomal localization^26^ and activation of mTORC1^21^, we hypothesized that lack of LAMTOR1 myristoylation and lysosomal localization would cause mTORC1 inhibition in cells with reduced NMT1 activity. First, we analyzed LAMTOR1 localization in cells with reduced levels of NMT1. We co-stained H460 control and NMT1 KD clones with LAMP1 and LAMTOR1 specific antibodies. Whereas LAMTOR1 co-localized with LAMP1 in 96.6 ± 3.1% of control cells, as expected (Fig. 5A), it was largely absent from LAMP1-positive lysosomes, with only 5.8 ± 1.3% of NMT1 KD H460 cells showing some co-localization. Transient transfection of NMT1 siRNA in H1792 cells also decreased LAMTOR1 lysosomal localization in most cells (55.4 ± 5.3% cells, Supplementary Fig. S5). Then, we treated cells with NMTi and analyzed LAMTOR1 and LAMP1 localization by IF. We observed that in control cells, LAMTOR1 and LAMP1 signals co-localized in 92.5 ± 5.3%, whereas only 4.1 ± 1.3% of H1792 cells treated with NMTi showed co-localization after 48 h of treatment (Fig. 5B), with LAMTOR1 being completely absent from the LAMP1-positive compartment in the entire cell population at 72 h after treatment in H1792 and H460 cells (Supplementary Fig. S5). LAMTOR1 was also absent from the LAMP1-positive compartment in HCT116, A375, HeLa and SKOV3 cells treated with NMTi (Supplementary Fig. S5).

Next, we tested mTORC1 localization in the presence of NMTi. We treated H1792 cells with vehicle control or NMTi (0.5 μM) for 72 h and stained them with LAMP1 and mTOR antibodies. Whereas mTOR preferentially co-localized with LAMP1 in 86.4 ± 3.4% of control cells, it was widely cytoplasmic in 80 ± 5.5% of NMTi-treated cells (Fig. 5C). These results confirmed that mTORC1 lysosomal localization in the presence of nutrients can be prevented using an NMT inhibitor, and provide an explanation for mTORC1 inactivation after NMT inhibition.

We also noticed a general decrease in LAMTOR1 immunofluorescence signal in H460 and H1792 cells treated with NMTi (Fig. 5B and Supplementary Fig. S5), which suggested decreased LAMTOR1 protein levels. To test whether total LAMTOR1 protein expression was affected by NMTi treatment, we performed WB analysis of NMTi-treated cells and observed a decrease (about 4-fold) in total LAMTOR1 protein levels as early as 24 h after treatment and sustained for up to 72 h in H1792 and H460 cells (Fig. 5D and Supplementary Fig. S5). Immunoblotting for mTOR in H1792 and H460 cells treated with NMTi indicated a moderate increase in total mTOR levels after 72 h of NMTi treatment (Fig. 5D and Supplementary Fig. S5), likely as a result of a compensatory feed-back mechanism elicited by mTORC1 inactivation.

Collectively, these findings indicate that sustained NMT1 activity is necessary for LAMTOR1-dependent lysosomal localization of mTORC1, and that lysosomal mTORC1 localization and activation can be effectively disrupted with a small molecule inhibitor of NMT in cancer cells.

### Silencing LAMTOR1 impaired lysosomal degradation and decreased colony formation in cancer cells

Next, we silenced endogenous LAMTOR1 using two different siRNA oligo sequences, which effectively decreased LAMTOR1 protein expression, and caused mTOR mis-localization in H1792 and HeLa cells, as expected (Supplementary Fig. S6).

**Figure 6:**
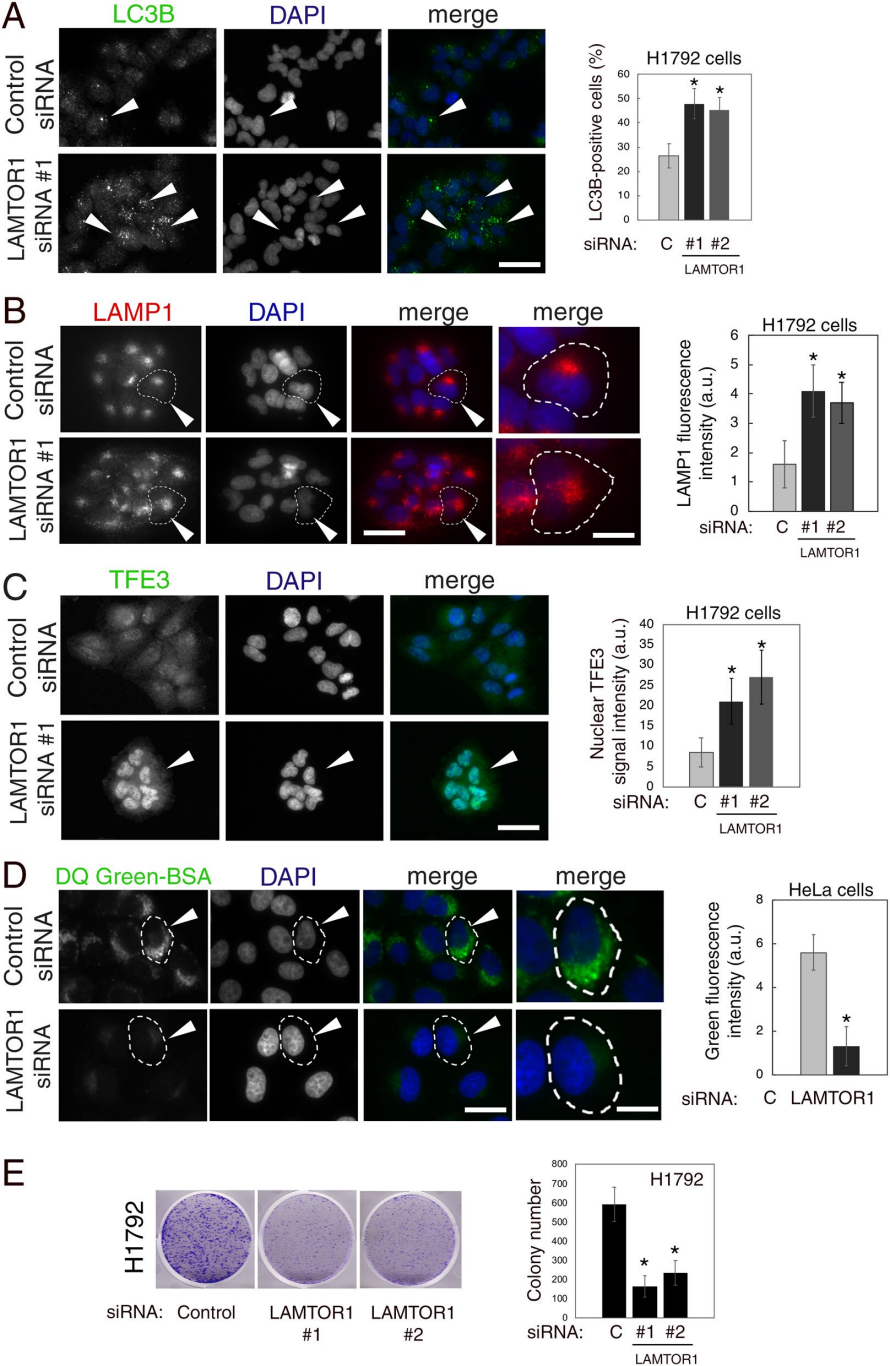
LAMTOR1 silencing decreased lysosomal degradation and impaired colony formation in cancer cells. (**A**) H1792 cells transfected with control or LAMTOR1 siRNAs for 72 h stained with an LC3B antibody and DAPI. Arrowheads, autophagosomes. Graph: LC3B-positive cells (at least 80 cells per condition and experiment) were quantified in two independent experiments. Results were combined and represented as average ± SEM. *p < 0.01 (Student’s *t* test). (**B**) H1792 cells transfected with control or LAMTOR1 siRNAs for 72 h stained with a LAMP1 antibody and DAPI. Arrowheads, outlined cell magnified in last column. Graph: LAMP1-positive signal intensity was quantified in two independent experiments (at least 25 cells per condition and experiment). Combined results are represented as average ± SEM. *p < 0.01 (Student’s *t* test). (**C**) H1792 cells transfected with control or LAMTOR1 siRNAs for 72 h stained with a TFE3 antibody and DAPI. Arrowhead, cell with strong nuclear TFE3 signal. Graph: Nuclear TFE3 signal intensity in two independent experiments combined (at least 20 cells per condition on each experiment) represented as average ± SEM. *p < 0.01 (Student’s *t* test). (**D**) HeLa cells transfected with control or LAMTOR1 siRNAs pool for 72 h incubated with DQ-Green-BSA to detect lysosomal degradation. DAPI, nuclei. Images show representative results from two independent experiments. Arrowheads, outlined cell magnified in last column. Graph: green fluorescent intensity per cell (at least 15 cells per experiment and condition) quantified in two independent experiments with similar results,  shown as average ± SEM for one representative experiment *p < 0.01 (Student’s *t* test). Bar, 5 μm in (**A**) and (**C**); 5 μm in (**B**) (columns 1–3) and 1 μm in (**B**) (last column); 1 μm in (**D**) (columns 1–3) and 0.25 μm in (**D**) (last column). (**E**) Colony assay in H1792 cells transfected with control or LAMTOR1 siRNAs for 6 days. Pictures, scans of representative wells. Three wells per condition were quantified in two independent experiments with similar results. Graph: colony number per well in one representative experiment, represented as average ± SEM. *p < 0.005 (Student’s *t* test) for each LAMTOR1 siRNA vs. control.

Similarly to NMT1 silencing, H1792 and HeLa cells depleted of LAMTOR1 showed accumulation of LC3B-positive autophagosomes (Fig. 6A and Supplementary Fig. S6), increased abundance of LAMP1-positive lysosomes/late endosomes (Fig. 6B and Supplementary Fig. S6), and increased nuclear accumulation of TFE3 (Fig. 6C). The above effects of LAMTOR1 silencing might be due to mTORC1 inactivation, but are also compatible with a defective autophagy flux. To investigate whether lysosomal degradation is affected by LAMTOR1 silencing, we incubated HeLa cells transfected with control or LAMTOR1 siRNA pool with DQ-Green BSA or AlexaFluor 549 BSA. Lysosomal degradation of DQ-Green BSA, but not global uptake of BSA, was impaired in cells depleted of LAMTOR1 when compared with cells transfected with a non-targeting control siRNA (Fig. 6D and Supplementary Fig. S6), suggesting that the inhibitory effects of NMT1 targeting on lysosomal degradation are also largely mediated by inactivation of LAMTOR1. These findings also indicate that LAMTOR1 is necessary to maintain lysosomal degradation and autophagy flux in cancer cells. Furthermore, LAMTOR1 silencing reduced colony formation in H1792 cells (Fig. 6E), suggesting that this lysosomal adaptor is necessary for proliferation and/or survival of H1792 cells.

Collectively, our results strongly suggest that NMT1 regulates lysosomal metabolic functions (mTORC1 activation and degradation) largely through modulation of the subcellular localization of the lysosomal adaptor and NMT1 target LAMTOR1. Accordingly, targeting the NMT1–LAMTOR1 axis using NMTi simultaneously inactivates mTORC1 and prevents lysosomal degradation, disrupting homeostasis and causing apoptosis in cancer cells.

### Pharmacological inhibition of NMT1 reduced tumor growth in a syngeneic mouse model of cancer

**Figure 7:**
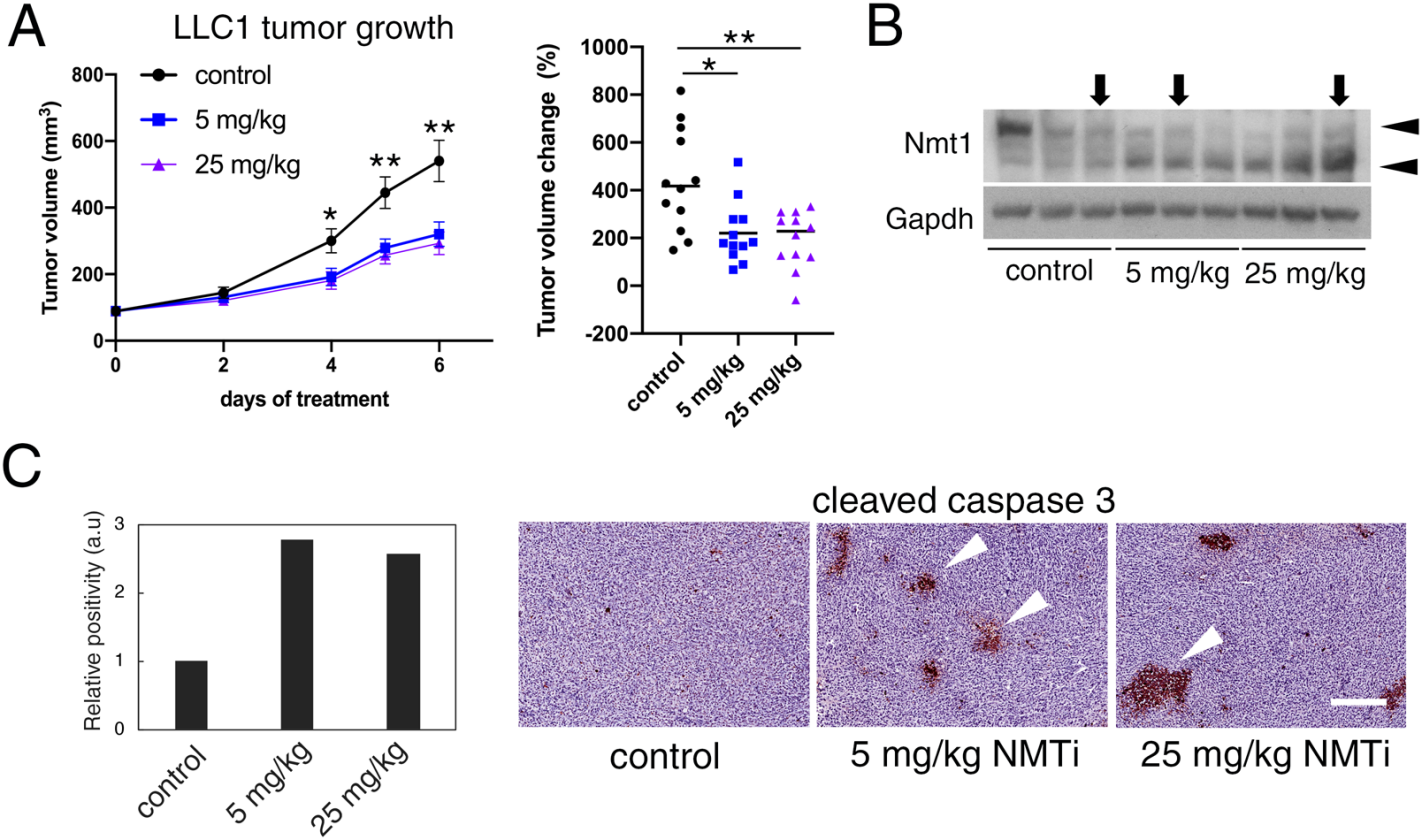
Decreased tumor growth and increased tumor apoptosis in mice treated with NMTi. (**A**) LLC1 cells were subcutaneously implanted into the same number of male and female C57BL/6J mice. When tumors reached an average size of ~ 90 mm^3^, animals were randomized into three groups (6 animals per group per experiment in two independent experiments) to receive vehicle control (5% DMSO in saline) or two different concentrations of NMTi (5 and 25 mg/kg) intraperitoneally daily. Data from both experiments were combined (12 animals per group). Left: tumor volume represented as average ± SEM. *p = 0.003 for both NMTi dosages vs control; **p = 0.002 for 5 mg/kg vs. control for days 5 and 6; and p = 0.001 for 25 mg/kg vs. control at days 5 and 6 (Student’s *t* test). Right, scatter dot plot representing percent tumor volume change for each individual animal belonging to the indicated experimental groups. Line, mean. *p = 0.0065 for 5 mg/kg vs control; **p = 0.0023 for 25 mg/kg vs control (Mann–Whitney test). (**B**) Tumors from three different animals per experimental group were analyzed by WB to detect Nmt1. Gapdh was used as a loading control. Arrowheads, full-length (upper) and cleaved (lower) Nmt1. Tumors from animals used in (**C**) were marked with arrows. Uncropped membrane scans are shown in Supplementary Figure S8. (**C**) Tumors corresponding to the indicated experimental groups were stained for cleaved caspase 3. Percent positive cells was calculated for the entire tumor section using Aperio software and represented normalized to control. Pictures correspond to representative regions of the analyzed tumors. Arrowheads, cleaved caspase 3-positive cells. Bar, 100 μm.

The effects of NMTi treatment at decreasing lysosomal degradation and simultaneously inhibiting mTORC1 suggest that this highly specific inhibitor of NMT could be efficacious against tumor growth in vivo. To test this hypothesis, we used a syngeneic model (Lewis lung carcinoma) in which subcutaneous tumors were generated upon injection of LLC1 cells into the rear flanks of wild-type immunocompetent mice. Once tumors were established, animals from both sexes were randomized into three experimental groups with an average tumor volume of 90 mm^3^. Groups received daily intraperitoneal injections of NMTi (5 and 25 mg/kg) or vehicle control (5% DMSO in saline) for the duration of the assay. Treatment with both NMTi dosages effectively decreased tumor growth, with a 2-fold reduction in mean tumor volume change (Fig. 7A). WB analysis of tumors protein extracts revealed cleavage of the Nmt1 protein in NMTi-treated animals (Fig. 7B), a known effect of apoptosis and NMT inhibition^12^.

Next, we analyzed paraffin-embedded tumor sections by immunohistochemistry (IHC) using an antibody against cleaved caspase 3 as a marker of apoptosis. The relative abundance of cleaved caspase 3-positive cells increased 2-fold in tumors from treated animals (Fig. 7C).

To analyze whether NMTi treatment had systemic adverse effects, organs (liver, kidney and spleen) from control and experimental animals were processed for H&E staining and histopathological examination. No differences were found among groups (Supplementary Fig. S7), indicating that NMTi treatment did not induce major toxicity to these organs under our experimental conditions. Furthermore, animal body weight followed a similar trend in control and experimental animals throughout the course of the assay (Supplementary Fig. S7).

Taken together, our findings indicate that the NMT inhibitor DDD85646 is efficacious at decreasing tumor growth in the presence of a functional immune system without inducing signs of systemic toxicity.

### Tumors from animals treated with NMTi displayed markers of lysosomal dysfunction

**Figure 8:**
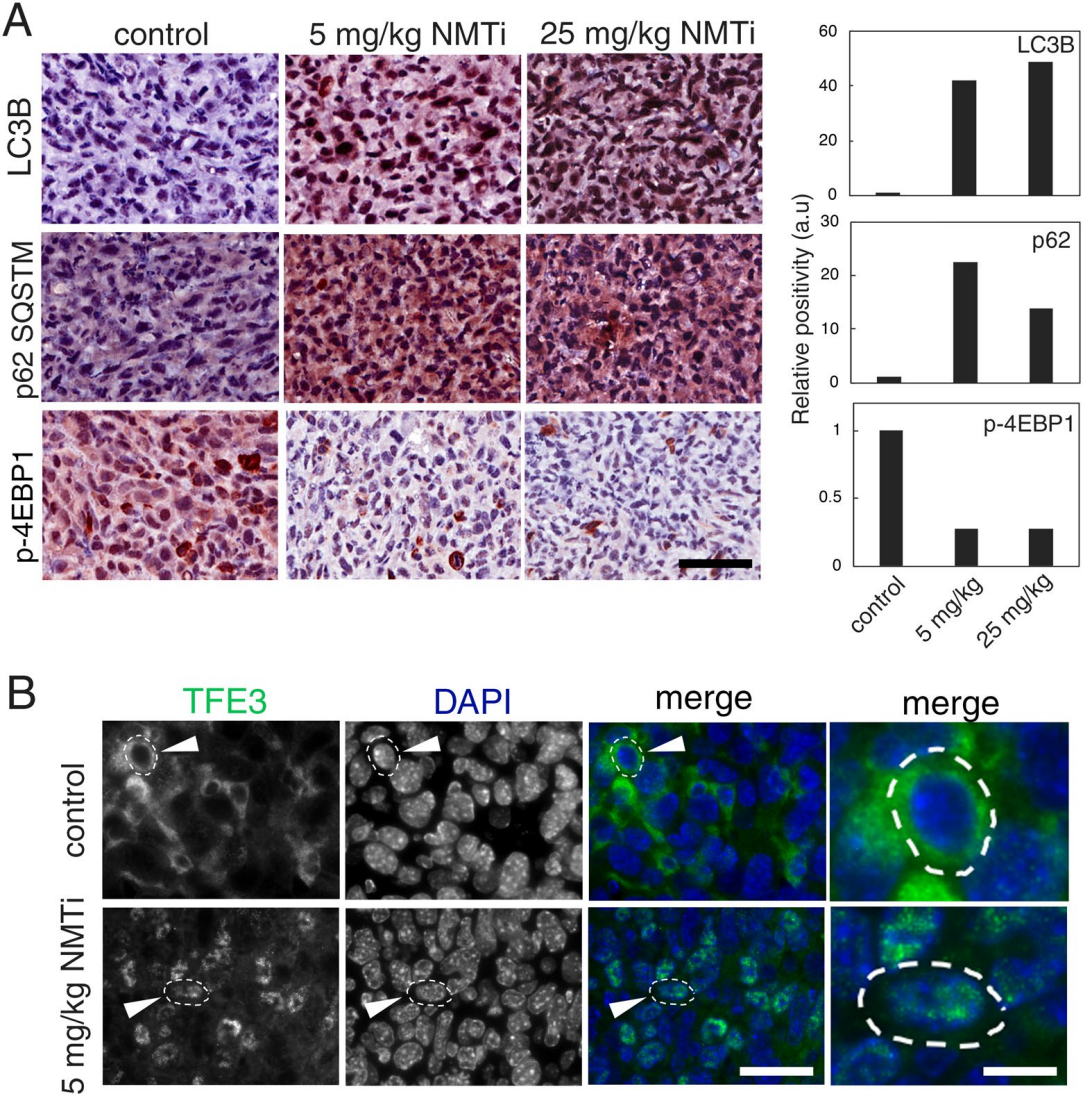
Markers of lysosomal dysfunction in tumors from mice treated with NMTi. (**A**) Tumors corresponding to the indicated experimental groups were stained for LC3B, p62, and p(T37/43) 4E-BP1. The percent positive cells was calculated for the whole tumor section using Aperio software, and represented as values normalized to control. Pictures correspond to representative regions of each tumor. Bar, 50 μm. (**B**) Tumors from control and 5 mg/kg NMTi-treated mice were stained with a TFE3 antibody and imaged using a fluorescent scope. Images are representative of the tumor sections (N = 2). Arrowhead, outlined cell magnified in the last column. Bar, 28 μm in columns 1–3 and 0.3 μm in last column.

To investigate whether tumors from NMTi-treated animals show signs of impaired lysosomal function, we evaluated the expression of autophagy-related proteins (LC3B and p62^SQSTM^), as well as markers of mTORC1 activation (phosphorylated 4E-BP1) in tumor sections using IHC. Tumors from treated animals had over 10-fold increase in LC3B and p62^SQSTM^ staining intensity, suggesting that autophagic flux might be compromised (Fig. 8A). In addition, phospho-4E-BP1 staining revealed a 5-fold decrease staining in tumors from NMTi-treated animals, indicating diminished mTORC1 activation. Finally, staining with a TFE3 antibody revealed nuclear accumulation of TFE3 in tumor cells (Fig. 8B). Our findings indicate the effectiveness of NMT1 inhibition at blocking lysosomal metabolic functions in vivo, and suggest that the NMT inhibitor DDD85646 represents a novel class of lysosomal targeting agent with promising therapeutic value in cancer.

## Discussion

In this study we report that NMT1 is an essential regulator of lysosomal metabolic functions in cancer cells. Inhibition of NMT1 impaired lysosomal degradation and inhibited mTORC1, leading to decreased cancer cell proliferation, increased apoptosis and decreased tumor growth. Our findings are in agreement with previous reports indicating that NMT1 is a cancer therapeutic target^9–13^, and support a general homeostatic function of NMT1 in cancer that is beyond oncoprotein myristoylation, and which could be exploited for therapeutic purposes in different cancer types.

Despite the number of target proteins susceptible of myristoylation by NMT1^44^, we found that the deleterious effects of targeting NMT1 on lysosomal function were largely attributed to the lysosomal adaptor LAMTOR1. However, based on our experiments we cannot completely rule-out that additional NMT1 targets contribute to lysosomal metabolic functions in cancer cells. Likewise, although NMT1 genetic targeting recapitulated most of the effects of NMTi treatment in lysosomal function, and we did not observe increased NMT2 expression in cells with reduced levels of NMT1, we cannot rule out that NMT2 plays a role in LAMTOR1 myristoylation, or lysosomal functions in cancer cells. Overall, the effects of genetic targeting of NMT1 were not as efficient as NMTi treatment in cancer cells, which might be explained by the fact that only a partial reduction of NMT1 expression was achieved by genetic targeting, likely due to the essential role of this gene in the cancer cells analyzed.

Our finding that NMT1-mediated myristoylation of endogenous LAMTOR1 is necessary for mTORC1 localization and activation is in agreement with previous studies^21^, and confirms that active myristoylation is necessary for the localization of endogenous LAMTOR1 to the endosome/lysosome compartment in cancer cells. It also indicates that mTORC1 is a novel effector of NMT1 in cancer cells. This is in agreement with the finding that mTOR inhibition increases NMT1 expression in breast cancer cells^47^, possibly due to a compensatory mechanism to increase LAMTOR1 myristoylation and restore mTORC1 activity in cells treated with mTOR inhibitors. We observed that NMTi treatment not only prevented LAMTOR1 localization, but also decreased total LAMTOR1 protein levels. Further investigation is needed to elucidate the specific mechanisms leading to decreased abundance of LAMTOR1 in the absence of NMT1 activity, but is is reasonable to speculate that it is due to protein degradation based on the presence of an N-degron in non-myristoylated LAMTOR1^48^.

We observed reduced LAMTOR1 lysosomal localization at 24 h after treatment with NMTi. However, phosphorylation of the mTORC1 substrates p70S6K and 4E-BP1 was not decreased until 48 h of treatment, suggesting that functional mTORC1 can be temporarily maintained at lysosomes in a LAMTOR1-independent manner, possibly through p62^SQSTM^ binding^49^.

mTORC1 inhibition is a well-known trigger of autophagy. However, mTORC1 inhibition after NMTi treatment caused a blockade of the autophagy flux due to impaired lysosomal degradation. Whereas we cannot rule out that mTORC1 inhibition by NMTi initially increases autophagy, the concomitant inhibition of lysosomal degradation ultimately blocked the autophagy flux causing aberrant accumulation of autophagosomes and defective lysosomes.

Interestingly, LAMTOR1 depletion was sufficient to inhibit lysosomal degradation in cancer cells, a result in agreement with a study in Lamtor1 null (p18^−/−^) mouse embryo fibroblasts^50^. These findings underscore the fact that LAMTOR1 has additional lysosomal metabolic functions beyond its role at tethering mTORC1 to the lysosomal surface and facilitating its activation by amino acids. The specific mechanism by which NMT1 and LAMTOR1 promote lysosomal degradation in cancer cells remains to be determined. In fibroblasts derived from lamtor1-null embryos, lysosomal degradation is impaired due to defective lysosomal-late endosome fusion^50^. Whether this or additional mechanisms are involved in cancer cells treated with NMTi remains to be investigated. Depletion of LAMTOR1 was reported to increase lysosomal abundance without decreasing lysosomal degradation in cancer cells^51^. This discrepancy might be due to differences in the models or the methodology employed.

We observed that LAMP1-positive vesicles appear more peripherally distributed in cells lacking NMT1 or LAMTOR1 that in control cells. Whether this is a passive effect due to increased lysosomal abundance, or is due to a more specific mechanism is currently unkown. The fact that Lamtor1-null mouse embryo fibroblasts and wild-type mouse embryo fibroblasts treated with mTORC1 inhibitors have similar lysosomal aberrant distribution without lysosomal accumulation^50^ indicates that the changes in lysosomal localization that we observe may be linked to NMT1-dependent regulation of mTORC1 activation.

Under conditions of nutrient availability, mTORC1 phosphorylation facilitates lysosomal localization of the transcription factor TFE3^36^. Under starvation or lysosomal dysfunction, TFE3 is dephosphorylated, translocates to the nucleus, and activates a lysosomal-initiated transcriptional program that includes expression of genes necessary for lysosomal biogenesis^34^. This regulatory mechanism is altered in some cancers, where TFE3 is constitutively nuclear^41^. Accordingly, we detect partial constitutive localization of TFE3 in the nucleus of HeLa and lung adenocarcinoma cells. Our finding that TFE3 further accumulates in the nucleus of cancer cells lacking NMT1, LAMTOR1, or in cells treated with NMTi is consistent with both the inhibition of mTORC1 activity by NMTi, and the autophagy blockade provoked by the lack of degradative lysosomal activity. It is possible that the increased lysosome abundance observed in cells lacking NMT1 or LAMTOR1, is the result of an attempt to restore lysosomal function through TFE3-induced lysosomal biogenesis. Consistent with this hypothesis, we observed that cells lacking TFE3 were more sensitive to die in the presence of NMTi treatment than those expressing TFE3. This is consistent with a role for TFE3 in tumor progression^52^, further indicating that TFE3 has unique functions in lysosomal homeostasis not fulfilled by TFEB. Despite the increased abundance in lysosomes in the absence of NMT1 or LAMTOR1, we found that these are largely non-functional.

Our experiments silencing LAMTOR1 indicate that most of the effects of NMT1 inhibition on lysosomal function are mediated through inhibition of LAMTOR1. We observed that LAMTOR1 silencing reduced colony formation, suggesting that LAMTOR1 is necessary for proliferation and/or survival of these cells. This is in agreement with previous findings showing p53-dependent apoptosis after LAMTOR1 depletion in cancer cells^51^. Most of the cells we used in this study are p53-deficient, suggesting additional pro-apoptotic mechanisms. Likewise, we cannot rule-out that additional NMT targets besides LAMTOR1, and additional mechanisms (such as ER stress^12^) are implicated in the pro-apoptotic effects of NMTi treatment.

The NMT inhibitor used in our study was originally discovered in a screen for trypanosomal NMT inhibitors^43^, and subsequently confirmed as a potent and specific human NMT inhibitor in studies mapping the human myristoylome^44^, comparing available NMT inhibitors^45^, and evaluating the compound’s effect in cancer cell proliferation, apoptosis and induction of ER stress^12^. While a single form of NMT exist in trypanosomes, mammalian cells have two isoforms (NMT1 and NMT2), which are similarly inhibited by this compound^44^. Whereas we cannot completely rule out a role for NMT2 in lysosomal metabolism, we observed that silencing of NMT1 was sufficient to recapitulate the effects of NMTi treatment, indicating that NMT1 is likely the main enzyme involved in regulating lysosomal functions in cancer cells.

Collectively, our results indicate that DDD85646 represents a novel class of lysosomal-targeting agent, which causes simultaneous inactivation of mTORC1 and blockade of lysosomal degradation, leading to cancer cell apoptosis. These therapeutically advantageous anti-cancer properties are shared with the quinacrine derivative compound DQ661, which targets lysosomal Palmitoyl-protein thioesterase 1 (PPT1)^42^.

Pharmacological inhibition of NMT1 using DDD85646 reduced tumor growth in vivo*,* and tumors from treated animals had increased apoptosis and expressed markers of lysosomal disfunction. Despite the roles of NMT1 and NMT2 in immune cell activation^6^, NMTi was effective at decreasing tumor growth in immunocompetent mice. Furthermore, NMTi treatment did not cause adverse systemic effects under the conditions tested, indicating a window of therapeutic opportunity.

In summary, we show that NMT1 is a novel regulator of lysosomal function in cancer cells, and that lysosomal degradation and mTORC1 activation can be simultaneously blocked by NMT inhibitors to limit tumor growth.

## Material and methods

### Cells

H460, H1792, HCT116, A375, HeLa, SKOV3, and LLC1 cells were obtained from ATCC and maintained at 37 °C and 5% or 10% CO_2_ in a humidified tissue culture incubator. Cells were grown in RPMI-1640 (H460, H1792, HCT116 and SKOV3) or DMEM (A375, HeLa and LLC1) obtained from Mediatech and supplemented with 10% Fetal Bovine Serum (Fisher). Cells were regularly tested for mycoplasm infection using MycoAlert mycoplasm detection kit (Lonza).

### General reagents and inhibitors

DDD85646 (NMT inhibitor) was purchased from Aeobius (AOB 6657) and used between 0.5 and 1 μM final concentration for most experiments. We verified batch purity by HPLC. Chloroquine was from MP biochemicals, DMSO was from Sigma-Aldrich, Crystal Violet was from Fisher. Puromycin and G418 were from Invivogen.

### Primary antibodies

NMT1 antibody was from Novus Biologicals (NBP2-32168) and NMT2 antibody from BD Transduction (611310). Antibodies against LC3B (clone D11), cleaved caspase-3 (9661), mTOR (clone 7C10), LAMTOR1 (clone D11H6), phospho-p70 S6 Kinase (Thr389) (9205), p70 S6 kinase (clone 49D7), phospho-4E-BP1 (Thr37/46), 4E-BP1 (clone 53H11), and β-Actin (clone D6A8) were from Cell Signaling Technology. p62^SQSTM^ antibody (18420-1-AP) was from ProteinTech. Antibody against TFE3 (HPA023881) was from Sigma Life Science. Antibody against LAMP1 (clone H4A3) to was from SouthernBiotech, and the antibody against GAPDH (clone 6C5) was from Millipore Sigma.

### RNAi

siRNA SMARTpools against human NMT1, LAMTOR1 and TFE3 were from Dharmacon, siRNA oligos #1 and #2 targeting human LAMTOR1 correspond to Dharmacon ON-TARGET plus individual oligos J-020916-17 and J-020916-19 respectively. Control non-targeting oligos were from Dharmacon. siRNA oligos were transfected in Opti-MEM using Lipofectamine RNAimax (Thermo Fisher Scientific), and experiments performed between 48 and 96 h after transfection. Effectiveness of KD was verified by WB on each experiment. Human NMT1 targeting shRNA (TRCN0000035710) and non-targeting shRNA control were purchased from Sigma-Aldrich as lentiviral transduction particles. Clonal selection by limited dilution was initiated 48 h after infection in the presence of 2.5 μg/ml puromycin.

### Immunofluorescence

Cells were grown on glass coverslips and fixed in 4% paraformaldehyde (Electron Microscopy Sciences) for 15 min. For LC3B and LAMP1 staining, cells were subsequently fixed for 5 min in ice-cold methanol. Cells were blocked and permeabilized using PBS containing 0.1% Triton X100 and 3% BSA for 1 h. Primary antibody was diluted in PBS containing 0.1% Triton X100 and 0.3% BSA and incubated overnight at 4 °C. After washing in PBS containing 0.1% Triton X100, cells were incubated with anti-rabbit or anti-mouse IgG conjugated with AlexaFluor 488 or AlexaFluor 594 (Thermo Fisher Scientific) (1:500) for 1 h. Coverslips were mounted in Vectashield containing DAPI (Vector Labs). Immunofluorescence images were acquired using a fluorescence AxioImager Zeiss microscope provided with a Zeiss AxioCam 503 camera and Zen Lite Software (Zeiss). For quantification experiments, between 5 and 15 images per coverslip were obtained randomly. Images were exported as “TIF” and quantified using ImageJ 1.52p (NIH) or Zen Software (Zeiss). Adobe Photoshop was used to assemble individual pictures into figure panels.

### Lysosomal degradation assay

H460 cells were incubated in RPMI containing 1% FBS in the presence of 0.5 μM NMT inhibitor or DMSO for 18 h and then labeled with AlexaFluor 594-BSA and DQ-green BSA (10 μg/ml each, Thermo Fisher Scientific) in RPMI containing 1% FBS in the presence of 0.5 μM NMT inhibitor or DMSO for 8 h. HeLa cells transfected with control non-targeting or LAMTOR1 siRNA pool were incubated in DMEM containing 1% FBS for 18 h and labeled with 10 μg/ml AlexaFluor 594-BSA for 1 h, or incubated in DMEM containing 1% FBS for 4 h and labeled with 10 μg/ml DQ-Green BSA for 18 h. After washing in PBS, cells were incubated in RPMI containing 1% FBS for 30 min, fixed in 4% PFA (Electron Microscopy Sciences), and mounted with Vectashield containing DAPI (Vector Labs). For quantification, images were randomly taken at 40 × magnification.

### Generation of TFE3-GFP cell lines

pEGFP-N1-TFE3 was a gift from Shawn Ferguson (Addgene plasmid # 38120)^33^. H460 and H1792 cells were transfected with 8 μg of purified DNA using Lipofectamine 2000 (Thermo Fisher Scientific). 48 h after transfection cells were selected in 1 mg/ml G418 and surviving pools expanded in culture in the presence of G418.

### Western blotting

Proteins were extracted using standard RIPA lysis buffer containing freshly added protease and phosphatase inhibitors. Frozen tumors were grinded and homogenized prior to lysis. Western blotting was performed using standard protocols. Briefly, membranes were blocked for 1 h at RT in 5% milk (BioRad) and incubated overnight at 4 °C with the indicated antibody dilutions prepared as recommended by manufacturer. After washing in PBS containing 0.1% Tween 20, membranes were incubated with horseradish peroxidase-conjugated anti-mouse or anti-rabbit IgG (GE Healthcare). Signal was developed using Supersignal WestPico PLUS (Pierce) and films scanned as digital images. Adobe Photoshop software was used for quantification of digital images.

### Myristoylation assay

Global myristoylation was measured following a established method^53^. Briefly, H460 cells grown overnight were washed with PBS to remove serum and pre-incubated for 1hour with Optimem (Thermo Fisher Scientific) containing 1 μM NMT inhibitor or DMSO control. This was then replaced by medium containing 1 μM NMT inhibitor or DMSO control and 25 μM of Alkynyl Myristic Acid (Click Chemistry tools) for 6 h in culture. Protein extracts were prepared using NP40-based lysis buffer and a total of 200 μg of protein per experimental condition assayed in the presence of biotin-PEG3-azide using a Click Chemistry Protein Reaction Kit as indicated by the manufacturer (Click Chemistry Tools). Pellet resulting from the reaction was resuspended in electrophoresis loading buffer, boiled and subjected to electrophoresis. Signal was detected with streptavidin-HRP staining (Cell Signaling Technologies) and developed with Supersignal WestPico PLUS (Pierce).

### Cell proliferation and colony forming assay

For proliferation curves, the same number of cells were plated in multiple plates, and treated 24 h after plating with the indicated amount of NMTi or DMSO control. At each time point, cells were collected by trypsinization and stained with Trypan Blue (Fisher). Viable cells were counted using a hemocytometer and mean values represented as a function of time. For colony forming assays, between 500–1,000 cells per well were plated in 6-well plates. Cells were treated with the indicated amount of NMTi or DMSO control 24 h after plating. After 6–10 days in culture, cells were stained with 0.1% crystal violet in 30% MeOH for 30 min at RT. Plates were scanned to digital images, and ImageJ used to quantify colony number.

### IC_50_ calculation

Cells were seeded in 384-well white plates (Greiner) at 375 to 1,500 cells per well according to the linear relationship measured from a standard curve of each cell line. 24 h after seeding, cells were treated with NMT inhibitor (three fold dilution, eight dilution points, in duplicate). After 72 h of treatment, cell viability was measured using CellTiter Glo Luminescent Cell Viability Assay (Promega), according to the manufacturer’s description, and IC_50_ values were calculated using the percentage of growth of treated cells versus the DMSO control with GraphPad Prism 7.0 software.

### Tumor generation and NMTi treatment in a syngeneic mouse model of cancer

All animal work was performed in strict accordance with an animal usage protocol approved by the Institutional Animal Care and Use Committee (IACUC) of The Lundquist Institute at Harbor-UCLA Medical Center (Institutional IACUC # 31364). C57BL/6J mice from both sexes (The Jackson Laboratory) were injected subcutaneously into the rear right flank with 2 × 10^6^ LLC1 cells resuspended in sterile PBS. When tumors were palpable, electronic calipers were used to measure tumor length (L), width (W) and height (H). Tumor volume was calculated using the formula (L × W × H)/2. Animals were randomized into 3 groups with a mean tumor volume of ~ 90 mm^3^, which received daily intraperitoneal injections of vehicle (5% DMSO in saline) or NMT inhibitor (5 and 25 mg/ml) for the duration of the experiment. Animal body weight was monitored daily. Animals were euthanized, and tissues harvested and immediately frozen in liquid nitrogen or fixed in 4% PFA. Tumor volume measurements from 2 independent experiments on a total of 12 animals (6 male and 6 female) per experimental group was analyzed according to sex and no differences were found between sexes. Data from both experiments were pooled to calculate mean tumor volume and percent volume change.

### Staining of paraffin-embedded mouse tumor and tissues

Tumors and organs from experimental animals were fixed in 4% PFA. Paraffin-embedding and H&E staining of tumors and organs was performed by the SBP Medical Discovery Institute Histopathology Core (La Jolla). Immunohistochemical staining was performed on 3 μm tumor sections following standard protocols. Briefly, endogenous peroxidase activity was blocked with 3% H_2_O_2_ (H325; Fisher Chemicals) in distilled water for 5 min. Heat-induced epitope retrieval was performed in a low pH citrate based Antigen Retrieval Buffer (MP-7401; Vector Laboratories) in a pressure cooker and sections were blocked with 2.5% horse serum. Primary antibodies were used at the following concentrations: cleaved caspase-3 (1:200), LC3B (1:700), p62/SQSTM1 (1:200), and phospho-4E-BP1 (Thr37/46) (1:1,600). All primary antibodies were diluted in Antibody Diluent (Agilent Technologies) and incubated for 1 h at room temperature except for LC3B which was incubated overnight at 4 °C. Signal was detected using ImmPRESS HRP Anti-Rabbit IgG Polymer Detection Kit and visualized with NovaRED Peroxidase (HRP) Substrate Kit (Vector Laboratories). Slides were counterstained with Gill’s No. 2 Hematoxylin Solution (GHS232; Sigma-Aldrich) and mounted with Cure Mount II (Electron Microscopy Sciences). TFE3 antibody was used at 1:500 overnight at 4 °C after heat-induced epitope retrieval in pH 6 citrate buffer. A secondary antibody conjugated with AlexaFluor 488 was incubated for 2 h a room temperature, and samples mounted in Vectashield containing DAPI. Automated Image Analysis: Slides were scanned at a magnification of 20X using Aperio Digital Scanning Systems (Leica Biosystems) at the UCLA Translational Pathology Core Laboratory. Acquired digital images representing whole-tumor sections were viewed and analyzed using ImageScope Analysis Software (version 12.4.0.5043; Aperio Technologies, Inc.). Tumor staining was quantitated by applying the “Positive Pixel Count v9” algorithm package to IHC and histochemical staining. To allow comparisons between tumor samples, the number of pixels with positive staining for NovaRED was expressed as relative to 100 pixels with positive staining for Hematoxylin (nuclei).

### Statistical analysis

Excel or Prism 8 software were used for statistical analysis. Statistical significance was calculated using Student’s *t* test (two sample assuming unequal variances) or Mann–Whitney test. Differences were considered significant when p < 0.05.

## Supplementary information

Supplementary Information 1.

## Data Availability

Materials and data generated during the current study are available from the corresponding author on reasonable request.
