# Supplementary material for: N-myristoyltransferase-1 is necessary for lysosomal degradation and mTORC1 activation in cancer cells
Source: Sci Rep. 2020 Jul 20;10:11952. doi: 10.1038/s41598-020-68615-w (PMC7371688; doi:10.1038/s41598-020-68615-w)
Supplement: Supplementary file 1 — Supplementary Information 1. [file 41598_2020_68615_MOESM1_ESM.pdf]

SUPPLEMENTARY INFORMATION

N-Myristoyltransferase-1 is necessary for Lysosomal Degradation and mTORC1 Activation in Cancer Cells

Yu-Chuan Chen, Marian S. Navarrete, Ying Wang, Natalie C. McClintock, Reiko Sakurai, Feng Wang, Kathryn T. Chen, Tsui-Fen Chou, Virender K. Rehan, Delphine J. Lee, and Begoña Diaz [1,*]

1. Department of Medicine, Division of Medical Hematology Oncology. The Lundquist Institute for Biomedical Innovation at Harbor-UCLA Medical Center. Torrance, California 90502, USA.

(*) Correspondence: bdiaz@lundquist.org

Supplementary Figure S1

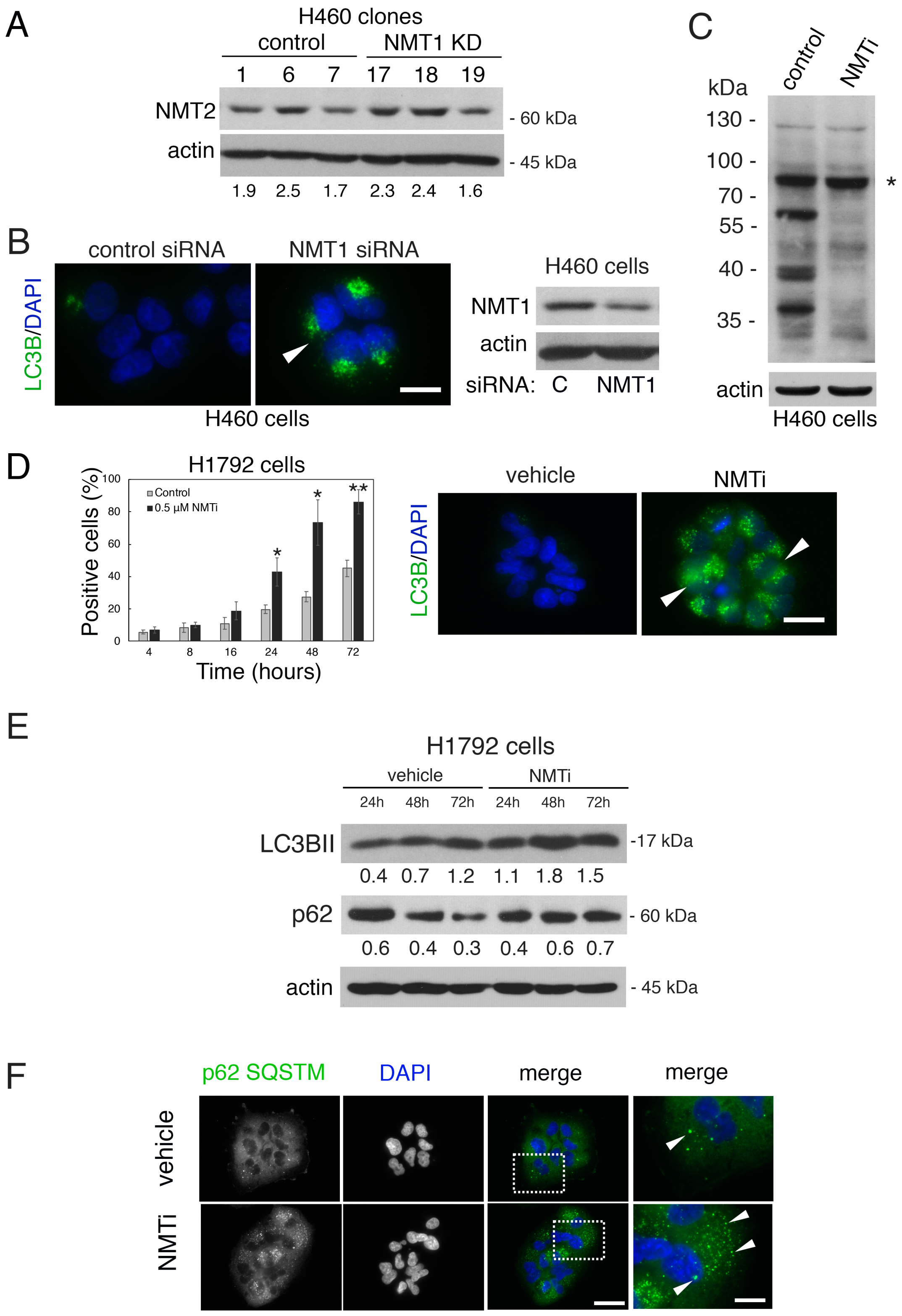

Supplementary Figure S2

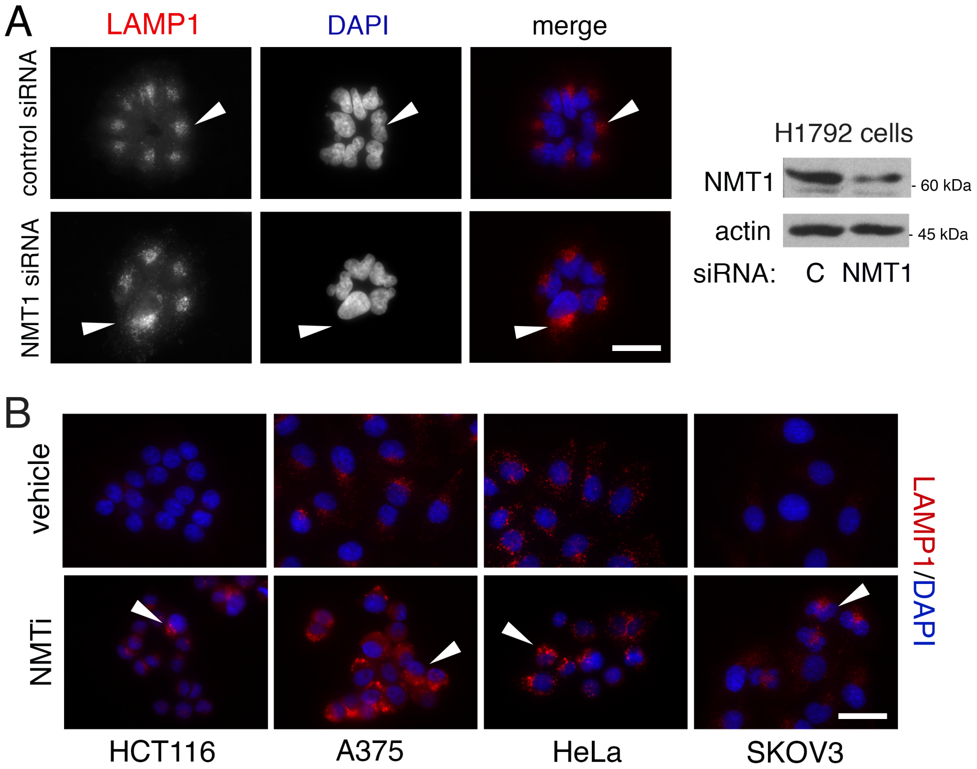

Supplementary Figure S3

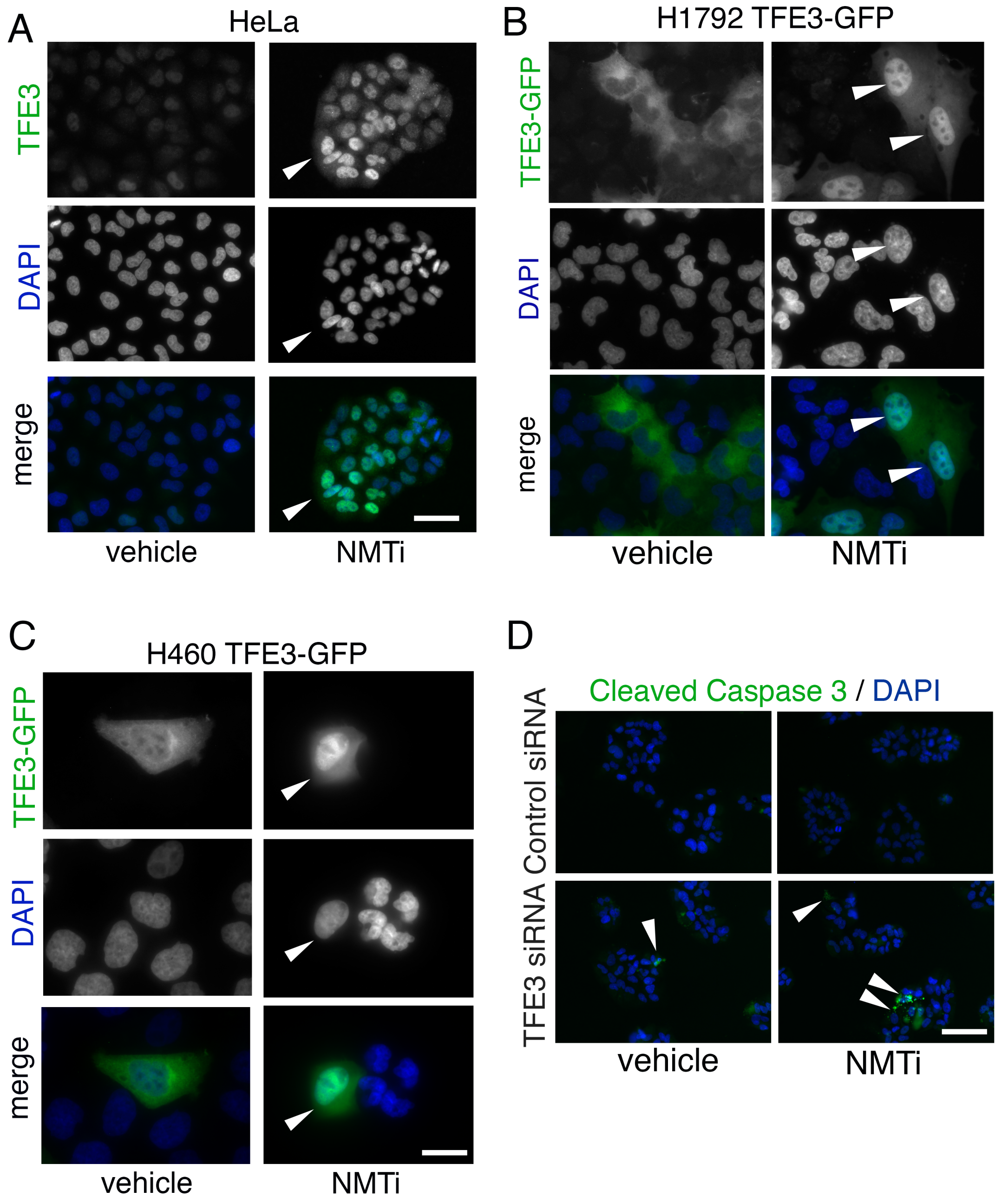

Supplementary Figure S4

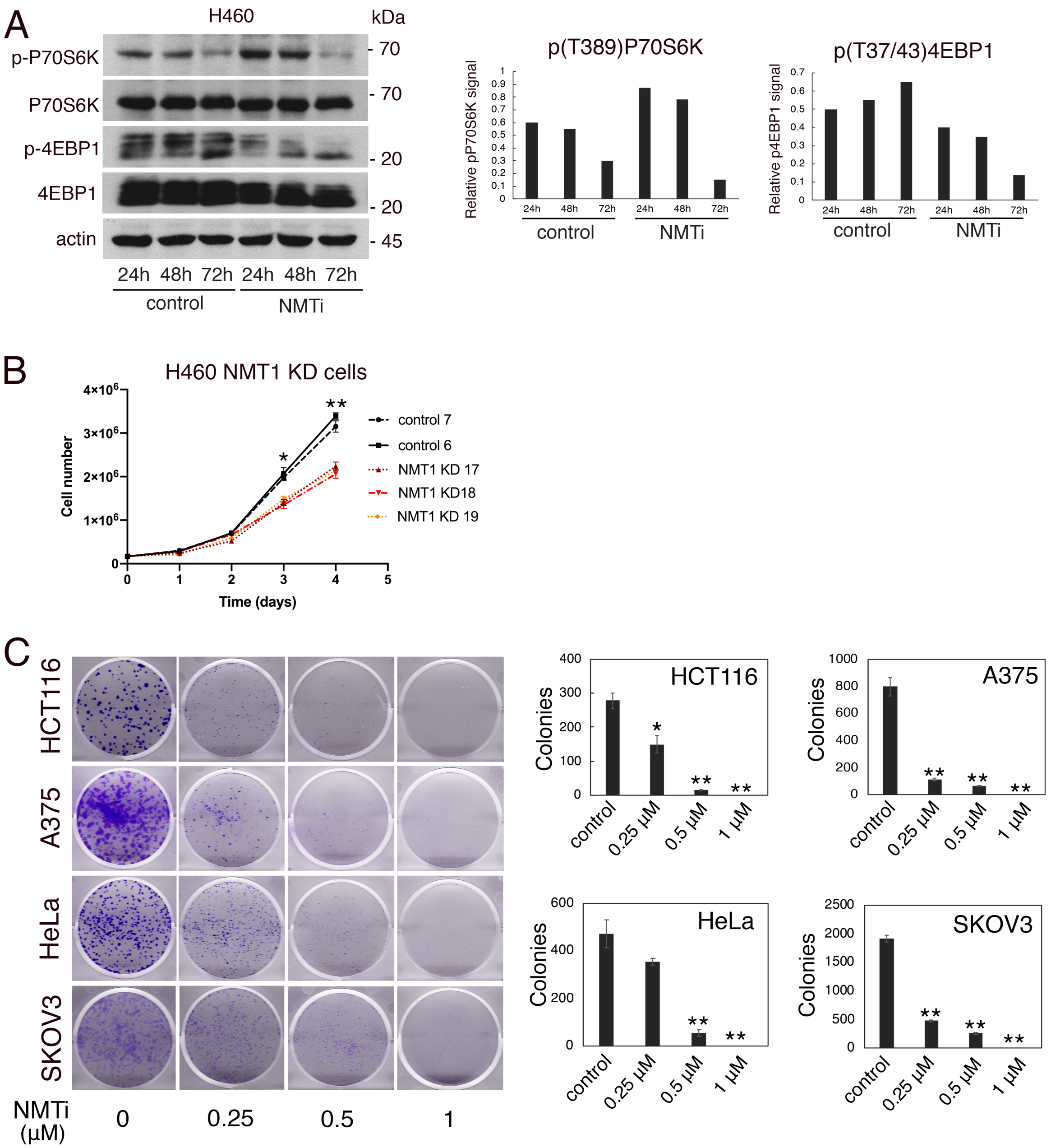

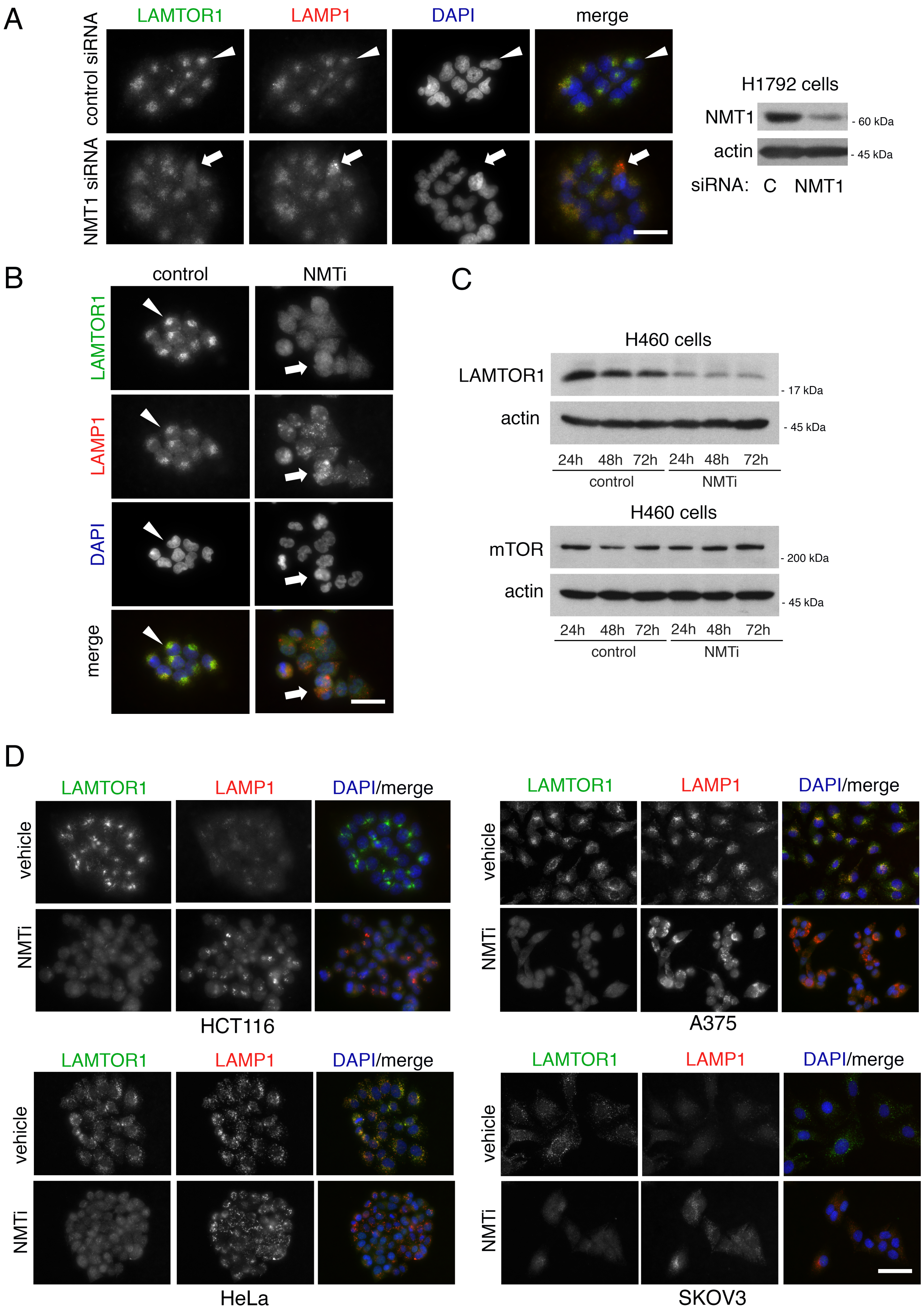
A
LAMTOR1
LAMP1
DAPI
merge
control siRNA
NMT1 siRNA
H1792 cells
NMT1
- 60 kDa
actin
- 45 kDa
siRNA: C NMT1
B
control
NMTi
LAMTOR1
LAMP1
DAPI
merge
C
H460 cells
LAMTOR1
- 17 kDa
actin
- 45 kDa
24h 48h 72h 24h 48h 72h
control
NMTi
H460 cells
mTOR
- 200 kDa
actin
- 45 kDa
24h 48h 72h 24h 48h 72h
control
NMTi
D
LAMTOR1
LAMP1
DAPI/merge
vehicle
NMTi
HCT116
A375
HeLa
SKOV3

Supplementary Figure S6

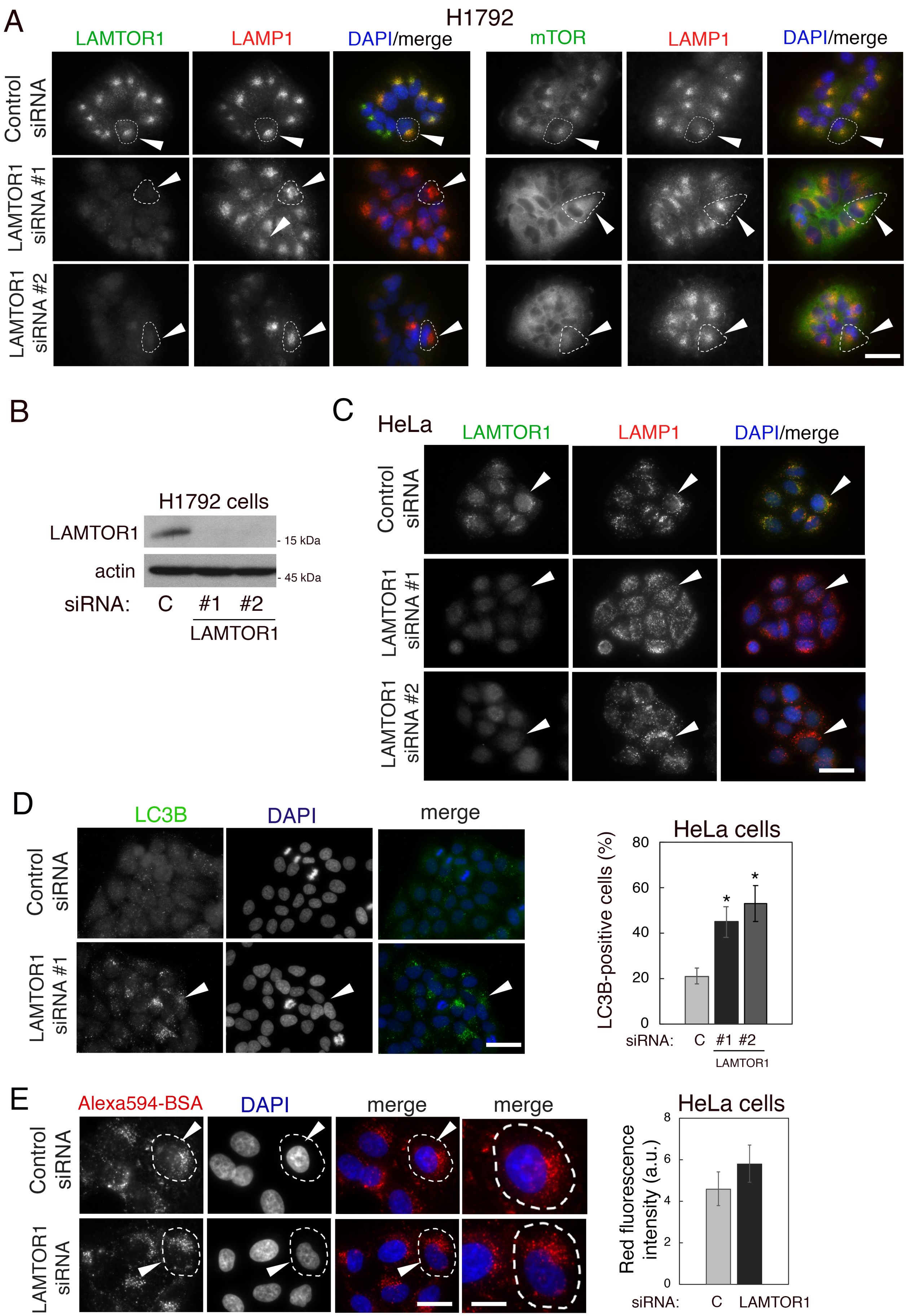

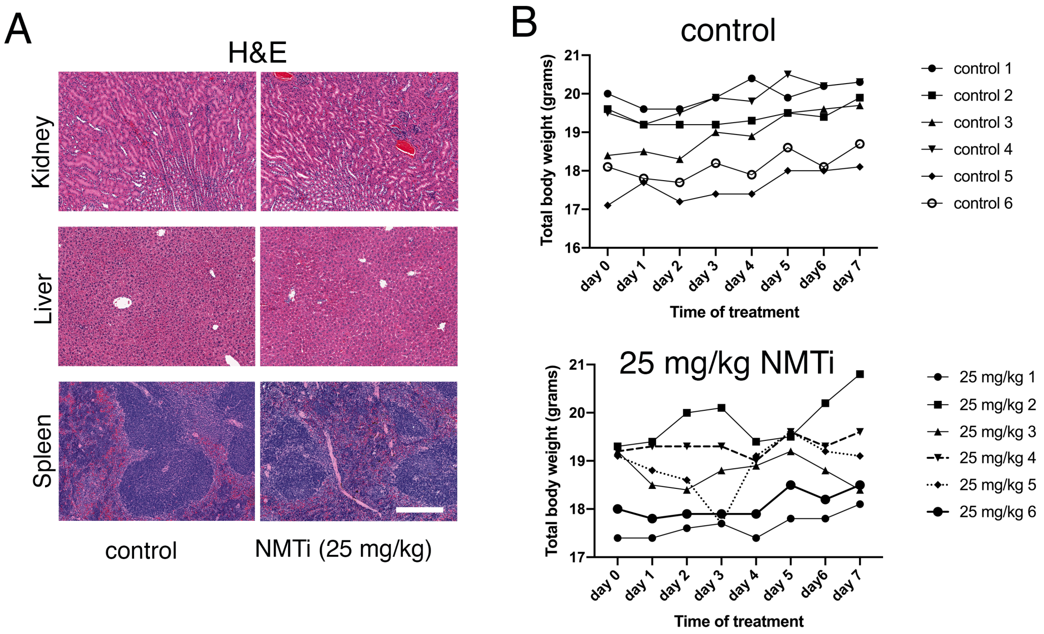
A
H&E
Kidney
Liver
Spleen
control
NMTi (25 mg/kg)
B
control
Total body weight (grams)
16
17
18
19
20
21
day 0
day 1
day 2
day 3
day 4
day 5
day6
day 7
Time of treatment
control 1
control 2
control 3
control 4
control 5
control 6
25 mg/kg NMTi
Total body weight (grams)
17
18
19
20
21
day 0
day 1
day 2
day 3
day 4
day 5
day6
day 7
Time of treatment
25 mg/kg 1
25 mg/kg 2
25 mg/kg 3
25 mg/kg 4
25 mg/kg 5
25 mg/kg 6

Uncropped Western blots

Figure 1

NMT1

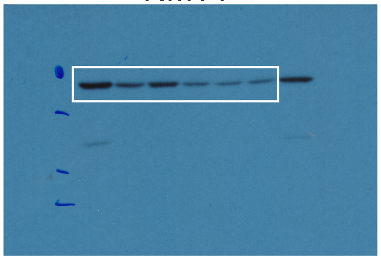

Actin

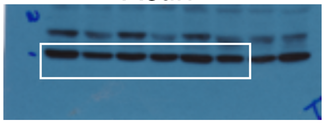

LC3B

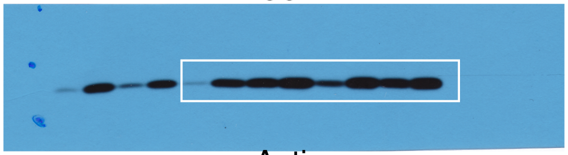

Actin

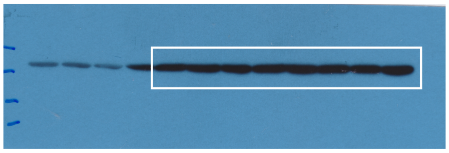

Figure 3

TFE3

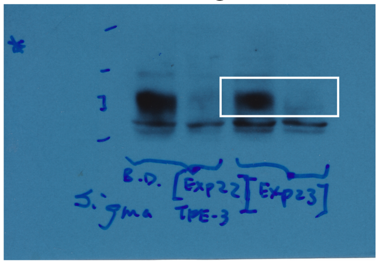

Actin

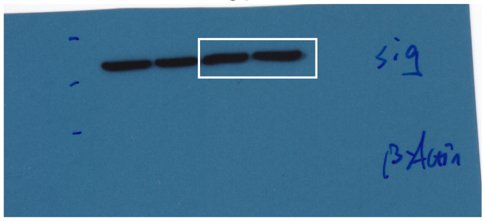

Figure 4

p-P70S6K

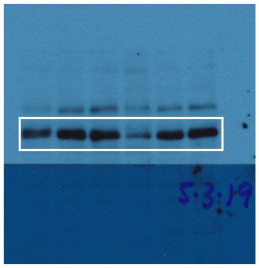

P70S6K

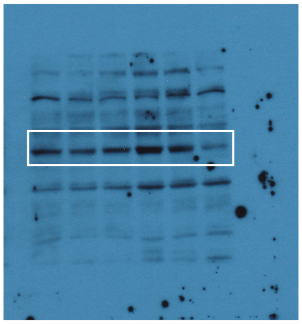

p-4EBP1

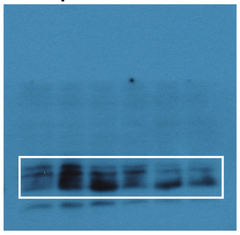

4EBP1

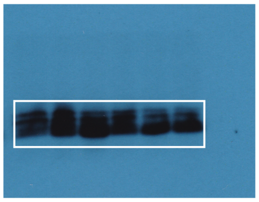

Actin

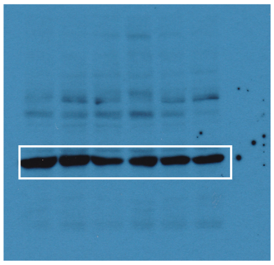

Figure 5

LAMTOR1

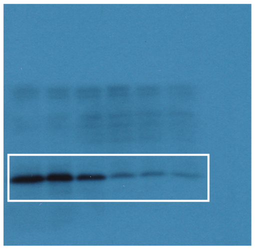

Actin

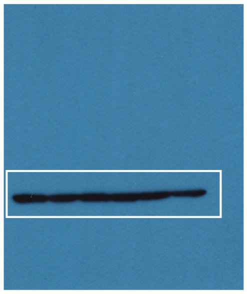

mTOR

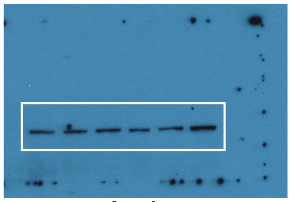

Actin

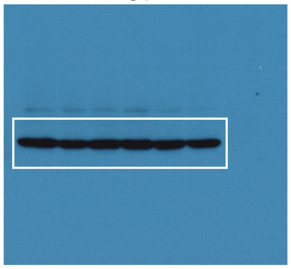

Figure 7

Nmt1

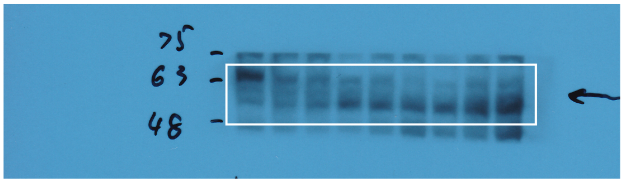

Gapdh

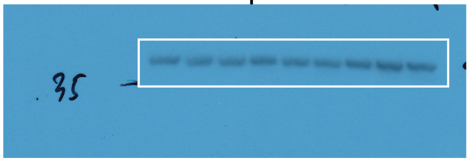

Uncropped western blots

Supplementary Figure S9

Suppl. Figure 1

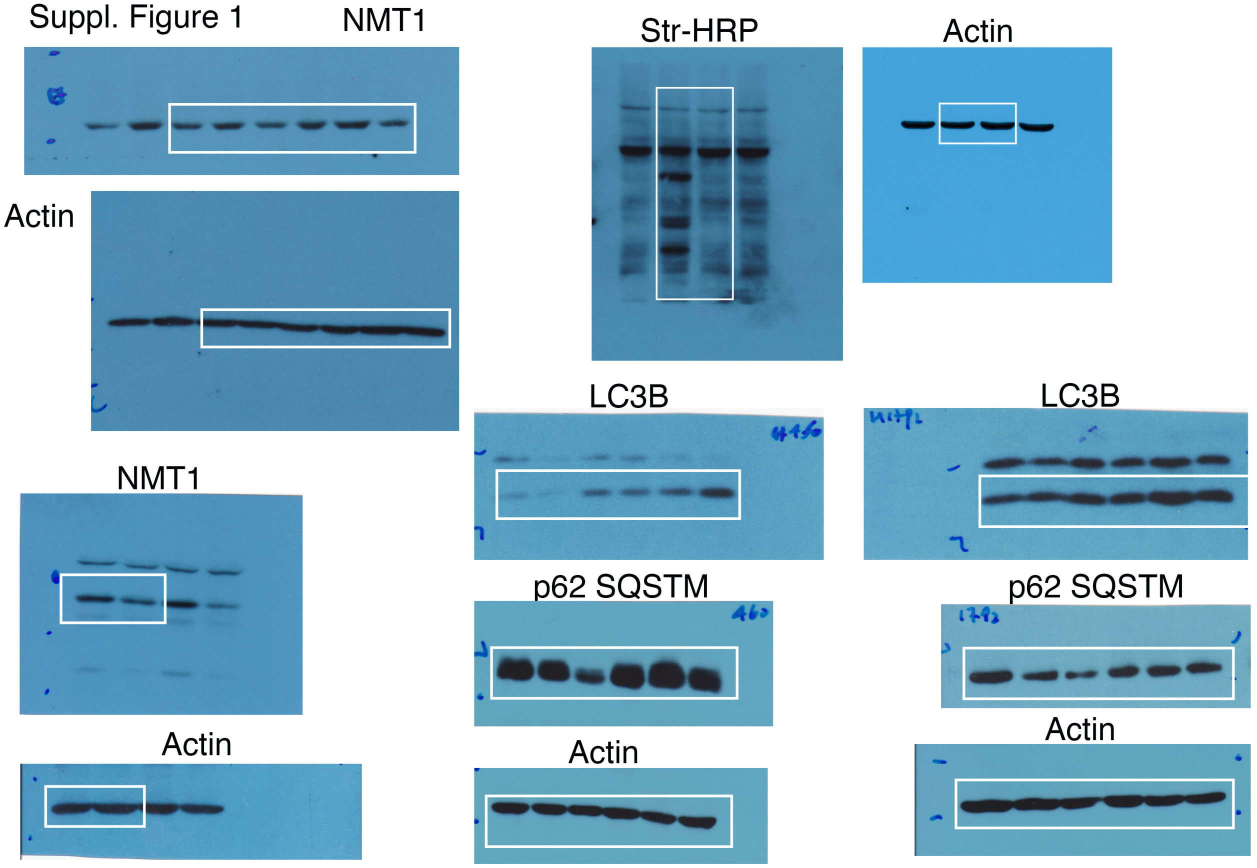

Suppl. Figure 2

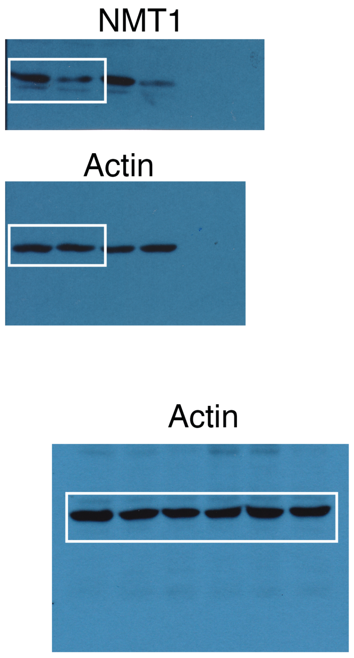

Suppl. Figure 4

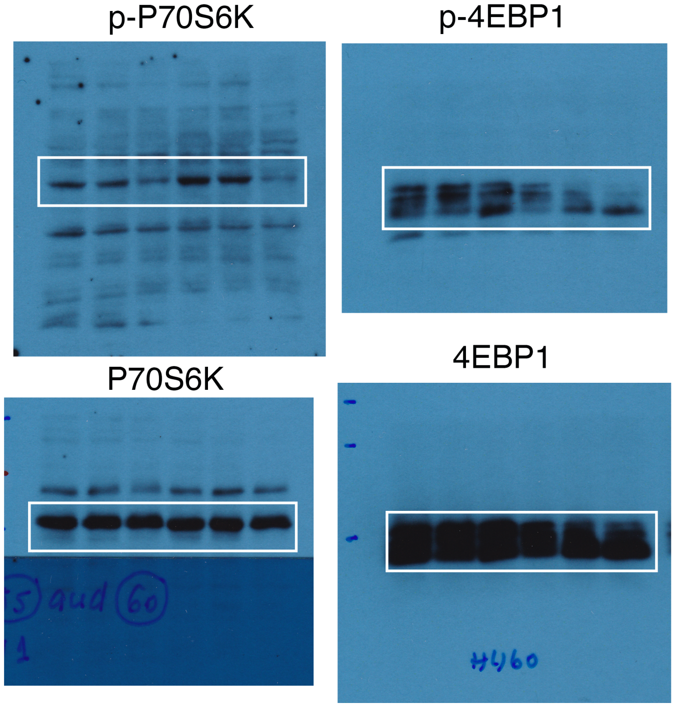

Supplementary Figure S10

Uncropped Western blots

Suppl. Figure 5

NMT1

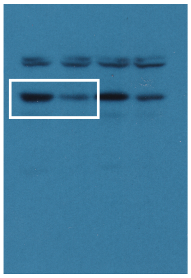

LAMTOR1

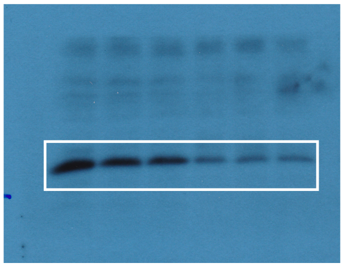

mTOR

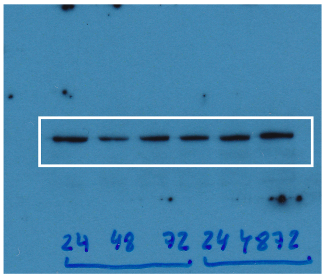

Actin

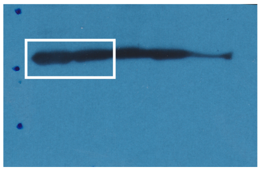

Actin

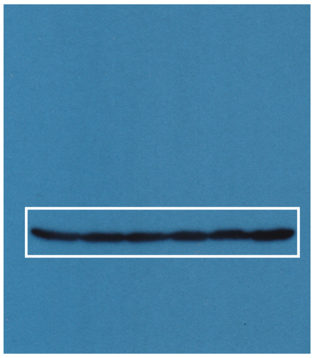

Actin

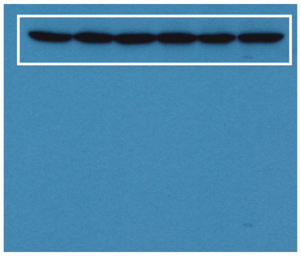

Supplementary Figure S1. NMT1 activity is necessary for the maintenance of autophagic flux in cancer cells. (A) WB for NMT2 in control and NMT1 KD H460 clones. Actin was used as loading control. Numbers under each lane are densitometry values (arbitrary units) for NMT2 signal normalized to actin in one representative experiment from two independent experiments with the same results. (B) Left, autophagosome detection by LC3B IF in H460 cells transfected with control and NMT1 siRNA and stained after 72 hours. DAPI was used to label nuclei. Arrowhead, autophagic vesicles. Bar, 2 μm. Right, WB analysis of NMT1 expression 72 hours after transfection. Actin was used as a loading control. Two independent experiments were performed with similar results. (C) Global myristoylation detection by click chemistry on lysates from H460 cells labeled with a myristate analogue and treated with control or 1 μM NMTi. Top: streptavidin-HRP immunoblot; bottom: actin re-probing. Three experiments were performed with identical results. (D) Autophagosome detection by LC3B IF in H1792 cells treated with DMSO control (vehicle) or 0.5 μM NMTi for the indicated times. DAPI was used to stain nuclei. Left, average percent value of cells containing LC3B-positive puncta from two independent experiments combined (at least 100 cells per condition and experiment were quantified). Error bars, SEM. (*) $p<0.01$, (**) $p<0.001$ (Student's t test). Right, representative pictures of LC3B and DAPI staining of H1792 cells treated for 72 hours. Arrowheads, autophagic vesicles. Bar, 3 μm. (E) H1792 cells were treated with 0.5 μM NMTi for the indicated times. WB results for LC3BII, $p62^{SQSTM}$ and actin (loading control) are shown for one representative experiment from two independent ones with similar results. Numbers under each blot are densitometry values (arbitrary units) for LC3BII and $p62^{SQSTM}$ signal normalized to actin. (F) H1792 cells were treated with 0.5 μM NMTi for 72h and processed for IF with a $p62^{SQSTM}$ antibody and DAPI to detect nuclei. Representative images from one experiments out of two with similar results is shown. Bar, 3 μm. Column 4, magnification of the area marked by a square in column 3. Bar, 1 μm.

Supplementary Figure S2. NMT1 targeting increased late endosome/lysosome abundance in cancer cells. (A) H1792 cells transiently transfected with non-targeting control or NMT1 siRNA pool for 72 hours were processed for IF to detect late endosome/lysosomes (LAMP1) and nuclei (DAPI). Representative images from one experiment are shown. Two independent experiments were performed with similar result. Right, NMT1 immunoblotting on cell lysates 72 hours after transfection. Actin was used as loading control. Arrowheads, LAMP1-positive signal in control and KD cells. Bar, 3 μm. (B) The indicated cell lines were treated with 1 μM NMTi or DMSO (vehicle) for 72 hours and processed for IF with LAMP1 and DAPI. Representative pictures from one experiment are shown. Two independent experiments were performed with similar results. Arrowheads, cells with increased abundance of LAMP1-positive vesicles. Bar, 3.5 μm.

Supplementary Figure S3. NMT1 inhibition caused nuclear accumulation of TFE3. (A) HeLa cells were treated with 0.5 μM NMTi or DMSO (control) for 72 hours and stained with a TFE3 antibody and DAPI. Arrowhead, cell with nuclear TFE3. Bar, 10 μm. The experiment was repeated twice with the same result. (B) H1792 cells stably expressing TFE3-GFP were treated with 0.5 μM NMTi or DMSO for 72 hours and stained with DAPI to identify nuclei. Images show representative results from two independent experiments. The phenotypes pictured were representative of over 90% of the cells in each experiment. Arrowheads, cell with nuclear TFE3-GFP. Bar, 3 μm. (C) H460 cells stably expressing TFE3-GFP were treated with 0.5 μM NMTi or DMSO for 72 hours and stained with DAPI to identify nuclei. Images show representative results from two independent experiments. The phenotypes in the pictures are representative of over 90% of the cells in each experiment. Arrowhead, cell with nuclear TFE3-GFP. Bar, 3 μm. (D) Representative images corresponding to data quantified in Figure 3D. H1792 cells transfected with non-targeting control or TFE3-specific siRNA pool were treated 24 hours after transfection with NMTi for 48 hours. Cells were stained with cleaved caspase 3 and DAPI to detect nuclei.

Supplementary Figure S4. NMT1 targeting decreased mTORC1 activation, cell proliferation and colony forming ability of cancer cells. (A) H460 cells were treated with 0.5 μM NMTi for the indicated times. Protein extracts were analyzed by WB using antibodies against p(T389)p70S6K and p(T37/43)4E-BP1. Antibodies against total p70S6K and 4EB-P1 were used for normalization and actin was used as loading control. Graph represents densitometry values (arbitrary units) for phospho- vs. total signal normalized to actin for one representative experiment from two independent experiments with similar results. Uncropped membrane scans are shown in Supplementary Figure S9. (B) H460 NMT1 KD cells were plated at the same density and treated with the indicated concentrations of NMTi. Number of viable cells was quantified at each time point (day 1 to day 4 after plating) in two independent experiments with similar results. Total number of viable cells per plate for each time point and condition are represented as average ± SEM. (*) $p<0.001$, (**) $p<0.0001$ (Student's t test) for each KD clone vs. both controls. (C) HCT116, A375, HeLa and SKOV3 cells were treated with the indicated concentrations of NMTi for 7-10 days and colonies stained with crystal violet. Pictures, scans of representative wells. Three wells per condition were quantified in two independent experiments per cell line with similar results. Right, quantification of colony number per well in one representative experiment, represented as average ± SEM. (*) $p<0.05$, (**) $p<0.0001$ (Student's t test) for treated vs. control.

Supplementary Figure S5. NMT1 is necessary for lysosomal localization of LAMTOR1 in cancer cells. (A) H1792 cells transfected with control or NMT1 siRNAs were stained with LAMTOR1 and LAMP1 antibodies along with DAPI. Panel shows representative fluorescence microscopy images of the indicated siRNA transfection. Two independent experiments were performed with identical results. Arrowheads, colocalization of LAMTOR1 and LAMP1 signals in control cells. Arrow, cell lacking LAMTOR1/LAMP1 co-localization in NMT1 siRNA transfected cells. Bar, 3 μm. Right, NMT1 immunoblotting on cell lysates 72 h after transfection was performed to verify KD efficiency. Actin was used as loading control. (B) H460 cells were treated with 0.5 μM NMTi or DMSO (vehicle) for 72 hours, and stained with LAMTOR1 and LAMP1 antibodies. DAPI was used to stain nuclei. Representative images are shown from one representative experiment. Two independent experiments were performed with the same results. Arrowheads, colocalization of LAMTOR1 and LAMP1 signals in control cells. Arrow, cell lacking LAMTOR1/LAMP1 co-localization in NMTi treated cells. Bar, 3 μm. (C) Protein lysates from H460 cells treated with 0.5 μM NMTi or DMSO (vehicle) for the indicated times were immunoblotted for LAMTOR1 and mTOR. Actin was used in each case as a loading control. Two independent experiments were performed with the same results. (D) The indicated cell lines were treated with 0.5 μM NMTi or DMSO (control) for 72 hours, and stained with LAMTOR1 and LAMP1 antibodies. DAPI was used to stain nuclei. Representative images are shown. Two independent experiments were performed with the same results. Arrowheads, LAMTOR1 at lysosomes in control cells; Arrows, NMTi-treated cells with LAMTOR1 mis-localization and increased LAMP1 staining. Bar, 10 μm.

Supplementary Figure S6. LAMTOR1 depletion mimics the lysosomal defects caused by NMT1 depletion. (A) H1792 cells were transfected with control or LAMTOR1 siRNAs #1 and #2 for 72 hours and processed for IF using LAMP1 antibodies in combination with LAMTOR1 (A) or mTOR (B). DAPI was used to mark nuclei. Representative images from two independent experiments are shown. Arrowheads, outlined representative cells magnified in the picture in last column. (B) Lysates from H1792 cells transiently transfected with control and LAMTOR1 siRNAs #1 and #2 were analyzed by WB 72 hours after transfection using a LAMTOR1 antibody. Actin was used as a loading control. (C) HeLa cells were transfected with control or LAMTOR1 siRNAs #1 and #2 for 72 hours and processed for IF using a LAMP1 antibody. DAPI was used to stain nuclei. Representative images from two independent experiments are shown. Arrowheads, representative cells in each image. (D) HeLa cells transiently transfected with control and LAMTOR1 siRNAs #1 and #2 were stained with an LC3B antibody and DAPI. Left, representative images for control and LAMTOR1 siRNA #1. Arrowheads, autophagosome accumulation in cells lacking LAMTOR1. Right, cells with LC3B-positive autophagosomes were quantified for each siRNA in two independent experiments (at least 30 cells per condition and experiment) and results represented as combined average ± SEM. (*) $p<0.01$ (Student's t test). (E) HeLa cells were transfected with control or LAMTOR1 siRNA pool for 72 hours and incubated with AlexaFluor 594-BSA for 3 hours. After fixation, cells were imaged to detect BSA uptake (red fluorescence). DAPI was used to label nuclei. Images show representative results. Arrowheads, outlined representative cells magnified in the picture in last column. Right, red fluorescent intensity per cell (at least 15 cells per experiment and condition) was quantified in two independent experiments with similar results. Graph shows average ± SEM for one representative experiment No differences were found in BSA internalization. Bar, 3 μm in A and C ; 10 μm in D; 1 μm in E (columns 1-3) and 0.25 μm in E (last column).

Supplementary Figure S7. NMTi treatment of mice did not affect histology of major organs or animal body weight. (A) Kidney, liver and spleen from animals treated with control and 25 mg/kg NMTi were stained for H&E and imaged. Bar, 100 μm. (B) Total body weight values for six individual animals bearing subcutaneous tumors per group (control and 25 mg/kg NMTi) is represented from the day of initial treatment (day 0) to endpoint (day 7).

Supplementary Figure S8. Uncropped western blot scans for main Figures.

Supplementary Figure S9: Uncropped western blot scans for Supplementary Figures.

Supplementary Figure S10: Uncropped western blot scans for Supplementary Figures.
